# Cookbook for plant genome sequences

**DOI:** 10.1186/s12864-026-12623-z

**Published:** 2026-02-06

**Authors:** Julie Anne Vieira Salgado de Oliveira, Nancy Choudhary, Samuel Nestor Meckoni, Melina Sophie Nowak, Marie Hagedorn, Boas Pucker

**Affiliations:** 1 Plant Biotechnology and Bioinformatics, IZMB, University of Bonn, Kirschallee 1, Bonn, 53115 Germany; 2 TU Braunschweig, Braunschweig, 38106 Germany

**Keywords:** Plant genomics, Long read sequencing, ONT sequencing, Pore-C

## Abstract

Access to genome sequences is crucial to investigate and engineer traits in plants, explore biodiversity, and support phylogenetic studies. Over the last decade, affordable sequencing devices have substantially increased the size of the genomics community by enabling individual research groups to sequence the genomes of plant species of interest. There has also been a quick development of novel tools for sequencing data analysis. Here, we share experiences with plant long-read genomics methods for Oxford Nanopore Technologies (ONT) and provide hands-on guidelines to support newcomers entering plant genomics. The full workflow from planning a plant genome sequencing project to the public release of associated data sets is covered.

**Supplementary Information:**

The online version contains supplementary material available at 10.1186/s12864-026-12623-z.

## Introduction

**Fig. 1:**
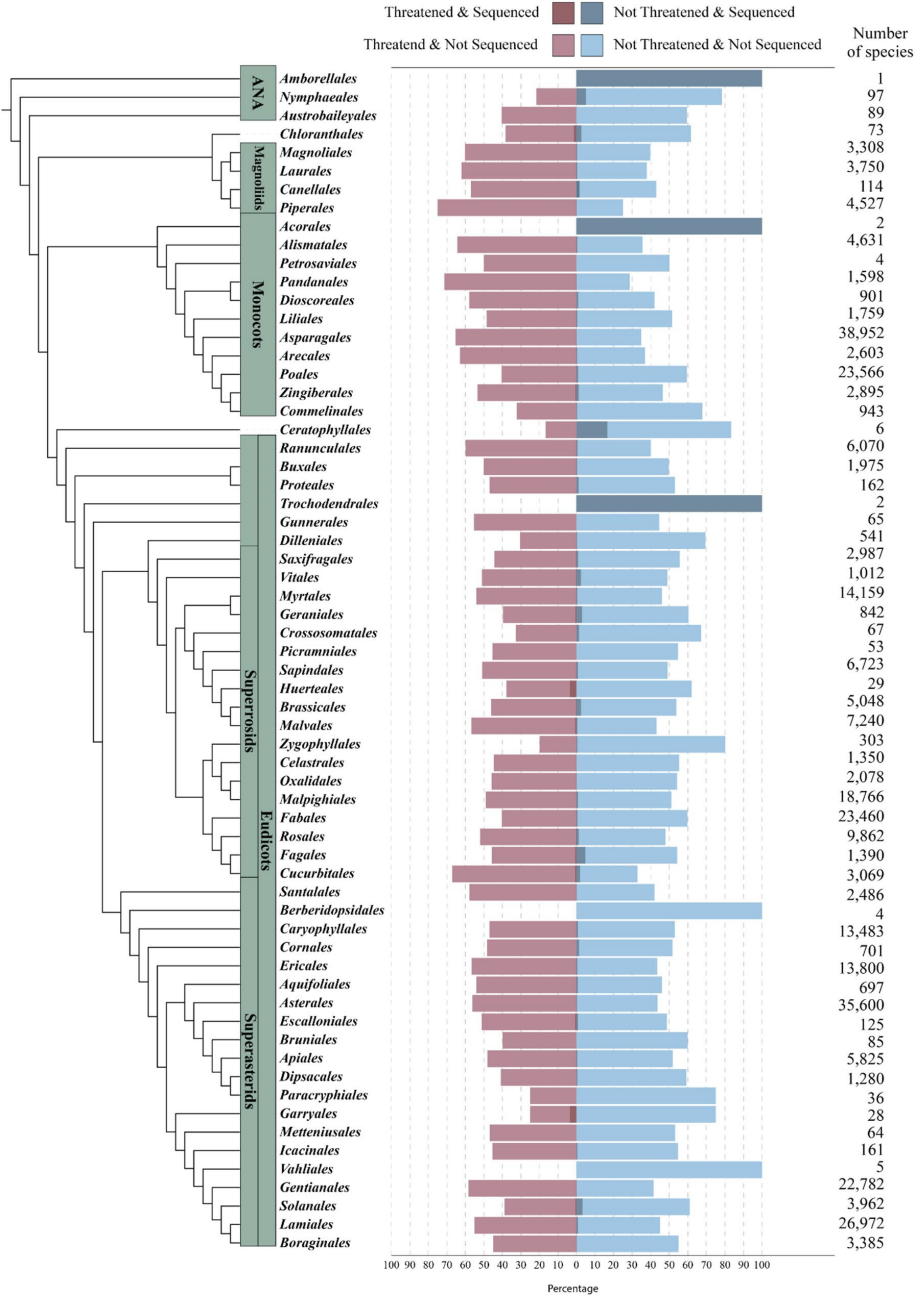
The percentage of threatened (left - in light red and dark red) and non-threatened (right - in light and dark blue) angiosperm species from Bachman et al. 2024 [12]. The species are grouped by order, representing the 64 orders of Angiosperms according to Angiosperm Phylogeny Group IV [13]. The darker red bars represent the threatened and sequenced species, while the darker blue bars represent the non-threatened and sequenced species. The numbers on the right represent the total number of known species in each order according to Bachman et al. 2024 [12]. Overall, based on angiosperm extinction predictions, around 45% of species are estimated to be threatened. In terms of genome sequencing, for the vast majority of angiosperm orders, less than 2% of species are sequenced, highlighting the dire need for conservation genomics prioritizing threatened species. The phylogenetic tree on the left is estimated from Janssens et al., 2020 [14], accessed using the Open Tree of Life [15]

The first ‘complete’ plant genome sequence, that of *Arabidopsis thaliana*, was released 25 years ago by a large international consortium as the result of an expensive process [1]. With the emergence and rapid improvement of long-read sequencing technologies, individual research groups can now generate *A. thaliana* genome sequences of superior quality [2, 3]. Currently, Pacific Biosciences and Oxford Nanopore Technologies (ONT) offer technologies for the generation of long and highly accurate sequences by analyzing individual DNA molecules [4]. We present a workflow that relies exclusively on ONT sequencing data and focus this review on this technology due to our specific expertise. ONT sequencing works by measuring changes in an electrical signal as a DNA strand passes through the nanopore. These changes in the electrical signal are caused by different nucleotide compositions that partially block the pore at any given moment [4]. Hundreds of plant genomes, including those of numerous crops, have been sequenced with long reads [4–6]. The ability to generate genome sequences for plant species of interest is crucial to harnessing the potential of orphan crops and crop wild relatives [5]. Engineering attempts, for example, those utilizing genome editing, can also profit from a high-quality genome sequence for experimental design [7]. As a single reference genome sequence cannot capture the full genetic diversity of a species, pangenome projects have been conducted to explore intraspecific diversity [8–10]. Despite all these efforts in plant genomics, we are still only seeing the tip of the iceberg. Based on an estimated number of 522,945 plant species [11] and 6,676 sequenced genomes accessible through NCBI, less than 2% of all plant genomes have been sequenced (Fig. 1). Given that multiple sequenced genomes may represent the same species, the actual proportion of covered plant species is likely considerably lower, highlighting significant opportunities for future genome sequencing initiatives. Due to legal restrictions (e.g., the Nagoya Protocol), not all scientists are allowed to study plant species native to biodiversity hotspots in the tropics. Therefore, it is important to enable local scientists to conduct genome sequencing projects before species become extinct, and their genomic resources are irreversibly lost.

To improve the coverage of plant species with available genome sequences, understanding the terminology associated with genome sequencing is crucial, as it highlights the differences between genomes and their in silico (i.e., computationally reconstructed) representations. This distinction is particularly important when interpreting scientific results and drawing conclusions about biological processes in nature based on these reconstructed sequences. A plant ‘gene’ is a segment of DNA present in a plant’s genome that contains the instructions for producing a specific protein or RNA molecule [16]. Unlike bacterial genes in polycistronic operons, plant genes also include regulatory regions, such as promoters, and often contain introns (non-coding regions), making their structure more complex [17]. In plant genes, coding sequences (CDSs) represent contiguous exons that encode proteins after intron splicing [18], distinguishing them from surrounding non-coding regulatory elements such as promoters, untranslated regions (UTRs), and introns. Many genes also contain UTRs that help regulate translation efficiency and mRNA stability. These non-coding elements play essential roles in gene regulation, allowing plants to respond to environmental changes. The gain, modification, or loss of these regulatory elements over time contributes to the diversity and adaptability of plant species [19, 20]. We define the term ‘plant genome’ as the complete set of DNA molecules present within a plant cell. From that, organellar DNA can be denoted as ‘subgenome’ and more specifically as ‘plastid genome’ or ‘plastome’ for the DNA from the plastids and ‘mitochondrial genome’ or ‘chondrome’ for the DNA from mitochondria. Consequently, ‘genome sequencing’ is the methodology employed to determine the order of nucleotides of this genetic material. Since a genome found in nature cannot be accessed with 100% accuracy, the in silico reconstructed sequence is designated as a ‘genome sequence’ or ‘assembly’. The term ‘assembly’ also refers to the process of combining smaller reads into larger sequences. Genomes typically consist of multiple distinct ‘chromosomes’. A high-quality assembly can accurately represent these chromosomes, which are then referred to as ‘pseudochromosomes’. A ‘Phred’ or ‘Q’ quality score is the probability that a nucleotide base is basecalled incorrectly in the sequencing data. When this score exceeds 20 (Q20+), it means that the probability of an incorrect basecall is less than 1%. The ‘coverage depth’ (hereafter ‘coverage’) refers to how many times a genomic region was read during sequencing, i.e., how many reads later include a specific position in the genome sequence. For example, if a genome is sequenced with 40x coverage, it means that, on average, each base is represented in 40 individual reads. Typically, for ONT sequencing, coverage less than 20x is described as low coverage, whereas around 30-50x is defined as moderate coverage. Higher coverage (more than 60x) is usually beneficial and sometimes necessary for large repeat-rich plant genomes [21]. In plants, the genome is usually diploid or polyploid. The various copies of one chromosome are termed ‘haplotypes’, while their in silico reconstructions are referred to as ‘haplophases’ [4]. The process of resolving haplophases is known as ‘phasing’. When haplophases cannot be resolved, for instance, due to the underlying organism being highly homozygous, this assembly is termed ‘unphased assembly’ and results in a single haplophase which represents both haplotypes.

**Fig. 2:**
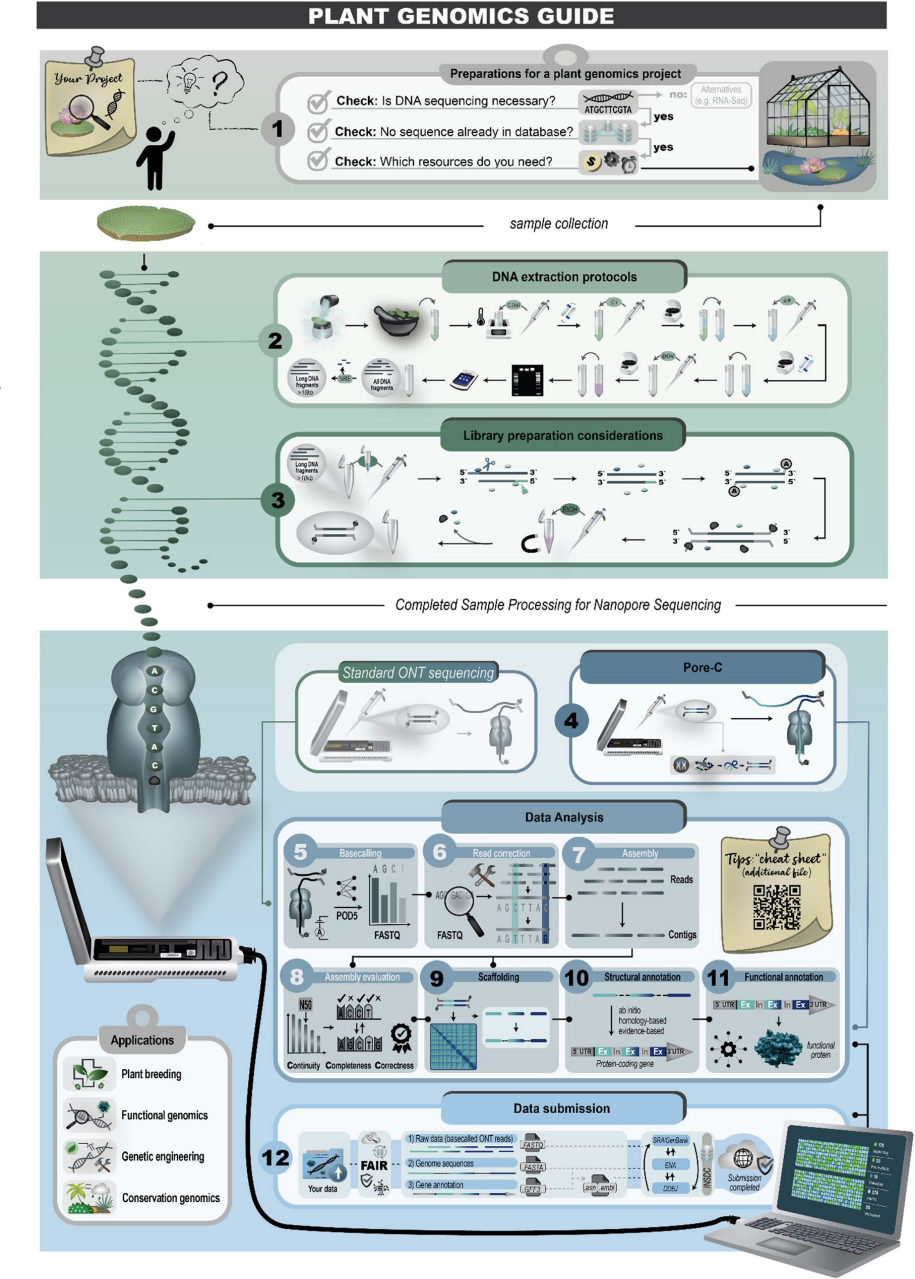
Major steps in a plant genome sequencing project using Oxford Nanopore Technologies (ONT). This workflow illustrates the twelve essential stages from project conception to data release. The process begins with assessing whether a new sequencing project is necessary, followed by sample collection and extraction of high-molecular-weight (HMW) DNA. Next, key library preparation considerations must be addressed before sequencing. An optional step involves chromosome conformation capture sequencing (e.g., Pore-C) to enable scaffolded assemblies. Sequencing output then undergoes a series of computational analyses: basecalling, read correction, genome assembly, and subsequent evaluation of assembly quality. Further steps include scaffolding, structural annotation, and functional annotation to generate a complete and biologically meaningful genome assembly. Finally, the resulting assemblies and associated data should be submitted to public repositories such as the INSDC databases. Genome sequencing has broad applications, including plant breeding, functional genomics, genetic engineering, and conservation genomics. To support newcomers, a hands-on guide for the data analysis steps is available as a supplementary file [22]

This review covers the major workflow steps, including preparing a genome sequencing project, DNA extraction, sequencing, genome sequence assembly, structural annotation, functional annotation, quality control for every step, and the submission of analysis results (Fig. 2). The guidelines and example commands shared here are intended to facilitate genomic sequencing projects conducted by individual research groups.

### Step 1: preparations for a plant genomics project

#### Importance of botanical gardens as a source of material for sequencing projects

In the age of plant genomics, botanical gardens are essential as repositories of a vast array of plant genetic diversity, being much more than simple displays of flora. These living collections contain a significant amount of the genetic diversity of plants worldwide and give scientists unparalleled access to a wide variety of documented plant material for genomic research [23]. When maintaining plant collections over long periods, botanical gardens inevitably maintain inbred or double-haploid plant lines, and these populations have high homozygosity, which can be beneficial for genome assembly and further analyses by reducing allelic variation and assembly ambiguities caused by heterozygous regions [24]. Examples of high-quality reference genome sequences utilizing haploid organisms or inbred lines are numerous [25–28]; homozygous genomes often lead to more contiguous and accurate reference assemblies. This homogeneity is especially advantageous because lower heterozygosity makes it possible to resolve structural variants and repetitive regions more clearly, which improves chromosome-level assemblies and facilitates functional genomics research [29–31].

The value of botanical garden collections in furthering genomic research is revealed as large-scale sequencing projects continue to progress. Advances in genome sequencing technologies are laying the foundation for large-scale projects like the Earth BioGenome Project. Specimens in botanical gardens are helpful resources for comparative genomics, as demonstrated in a study by Liu et al. in 2019, which generated sequences of nearly 700 vascular plant species from the Ruili Botanical Garden [32]. Several of our studies began with plants acquired from botanical gardens and would not have been possible without these valuable resources. Examples are the water lily *Victoria cruziana* acquired from TU Braunschweig Botanical Garden that was used to generate a high-quality genome sequence [33]; the cacao study [34] that enabled the investigation of the genetic basis of flavonoid biosynthesis, and the discovery of the withanolide biosynthetic gene cluster in the *Withania somnifera* genome sequence [35].

Botanical gardens can be considered living bridges between taxonomy, conservation, and genomics, and will continue to be essential for plant science and for safeguarding the genetic repertoire of the world’s flora.

#### Evaluating existing genomic data

Before initiating a sequencing project, it is essential to assess whether genome sequencing is necessary. In certain applications, RNA-seq data may be sufficient to assemble sequences of expressed protein-coding genes, which can provide adequate information for specific projects [36, 37]. The required RNA-seq data sets may already be available in a public database. If genomic reads or a complete genome sequence are required, the subsequent step involves determining whether a genome sequence of sufficient quality is already available. This question can be answered by consulting databases maintained by members of the International Nucleotide Sequence Database Collaboration (INSDC), including GenBank and the Sequence Read Archive (SRA), the European Nucleotide Archive (ENA), and the DNA Data Bank of Japan (DDBJ) [38]. Since some of these databases offer reads in addition to genome sequences, it is essential to search for both assembled genome sequences and raw reads. Repositories hosting reads include the SRA and ENA. Accessing reads can facilitate projects by bypassing the sequencing step and allowing for the immediate start of dry lab work. Other big databases are Phytozome and the National Genomics Data Center (NGDC) [39, 40]. Additionally, a simple internet search may be helpful, as other specialized databases or data repositories like FigShare [41], Dryad [42], e!DAL [43] and PGP [44] exist, and some authors may make their data available outside major databases.

#### Resource estimation utilizing the plant DNA C-values database

To prepare for the sequencing of a plant genome, it is essential to estimate the required resources, which are mostly dependent on the genome size. A preliminary assessment of the genome size can be achieved using the C-value, which represents the amount of DNA in picograms per haploid nucleus and can be obtained from the Kew Garden Plant DNA C-value database, which, in its current release (Release 7.1, April 2019), contains data for 12,273 species [45].

Resource needs are determined by the project’s specific aims and the characteristics of the plant material, both of which affect the volume of sequencing data required. The availability of homozygous or double-haploid organisms is often limited, unless the plant of interest is subject to extensive cultivation or breeding efforts. For instance, the sugar beet reference genome sequence (RefBeet) was established using a double haploid line to reduce assembly complexity [46]. The study by Shi et al., 2023, provided a complete reference genome sequence for grapevine, using the PN40024 line (from the cultivar Helfensteiner) which originated from self-fertilization carried out for nine generations, resulting in a 99.8% homozygous genome [47]. When such resources are inaccessible, a haplotype-resolved genome assembly becomes necessary, effectively doubling the data requirements based on the C-value. This demand may further escalate if the genome of the plant in question is polyploid, which is a very common property of plant genomes [4, 48]. In our experience, a coverage depth, i.e., the number of reads spanning each genomic position, of 30x per haplophase was sufficient to obtain high continuity assemblies. This is also recommended by others for haplotype-resolved assemblies [49, 50]. While the initial calculation based on biochemical data, like a C-value, is only a rough estimate, the first sequencing data can be used for a genome sequence assembly attempt that allows a more accurate assessment of the actual obtained coverage. This analysis can be repeated as more data become available to ensure that a sufficient amount of sequencing data is generated. Generating more data than needed based on the coverage recommendation above should generally improve the assembly quality. By using the full capacity of committed flow cells, it might be possible to generate this additional data by just letting the run continue until all nanopores are broken or blocked.

For example, *Victoria cruziana* has a 1 C value of 4.10 [51], indicating the need to obtain a total of 123 gigabasepairs (Gbp) of long read sequencing data. Following a general guideline suggesting an average yield of about 10 Gbp per MinION flow cell, at least 12 flow cells are estimated to be necessary. Additionally, further read correction processes may lead to a decrease in the overall yield of final sequencing data that can be subjected to the assembly process. The data output heavily depends on the DNA quality, which can be influenced by the quality of the individual plant material. Therefore, it is imperative to optimize the DNA extraction process to ensure optimal sequencing results.

Another alternative for estimating the genome size, when (short) reads are already available, is k-mer counting. A k-mer is a substring of length k. A k-mer-based genome size estimation utilizes the frequency of read k-mers. Tools like Jellyfish [52] and KMC [53] can be used for the calculation of k-mer frequencies, while GenomeScope 2.0 uses the k-mer frequency distribution with statistical modelling to estimate genome characteristics such as size, heterozygosity, and repetitiveness [54].

### Step 2: DNA extraction protocols

The fundamental step in plant genomics is the extraction of high molecular weight DNA to obtain long sequencing reads that enable the resolution of complex and/or repetitive genomic regions [55, 56]. Since the sequencing technology imposes no limit on the read length, the read length distribution depends directly on the DNA fragment size subjected to the sequencing. Extracting DNA from plants is challenging due to particularities such as the cell wall, starch, and other polysaccharides, and specialized metabolites like polyphenols [57]. With the introduction of the Short Read Eliminator (SRE) kit (originally by Circulomics), it became critical to ensure the presence of long DNA fragments in the sample, while the depletion of short fragments can be reliably performed in a final step using the SRE kit.

Over the years, a wide range of optimized protocols has been developed, including cetyltrimethylammonium bromide (CTAB)-based methods, which have been widely adopted by the plant science community due to their effectiveness with diverse plant species [58]. This method begins with the disruption of the tissue in liquid nitrogen, followed by grinding into a fine powder with a pestle and mortar. The tissue is then treated with a CTAB buffer, which facilitates cell lysis and prevents specialized metabolites from interfering with the DNA [59]. Customized CTAB protocols can have several modifications to address different challenges faced with material from different plant species, since plants differ substantially in their content of sugar, acids, or specialized metabolites. An example of effective modifications that provide high molecular weight DNA for long read sequencing was applied in the study of Siadjeu et al., 2020, with an SRE kit to enhance long DNA fragments [34, 60]. The use of fresh plant material is essential, and young leaves are recommended due to a high ratio between the DNA-containing nuclei and metabolites accumulated in the central vacuole. Keeping plants in the dark before extraction is recommended to prevent starch accumulation and reduce plastid content [61].

For some challenging plants, it may be necessary to perform nuclei isolation prior to DNA extraction to reduce the complexity of the biological system, such as the presence of specialized metabolites. Additionally, nuclei isolation can help reduce the amount of organelle DNA in the final sample [62–64]. This type of protocol involves disrupting cell walls and membranes while preserving nuclear integrity, followed by the isolation of nuclei using density gradient centrifugation or filtration methods [63, 65–67]. Nuclei isolation has been shown to be especially beneficial for long-read sequencing technologies that require high-molecular-weight DNA, with applications demonstrating that nuclei-based DNA extraction protocols can produce larger DNA fragments with fewer contaminants [64, 67–70]. Another notable method is magnetic disk DNA extraction, which uses a silica-coated magnetic disk to isolate DNA through standard lysis, binding, washing, and elution steps. This protocol is recommended for applications that require high molecular weight DNA, as the disk-binding mechanism allows DNA to bind and release with minimal fragmentation [71–73].

### Step 3: library preparation and sequencing

Library preparation is a set of steps that converts purified nucleic acids into a collection of fragments ready for sequencing. In our hands, a ligation-based approach was most successful in generating data for plant genome assemblies. The process involves repairing the DNA before adapter ligation. An alternative approach aims at preserving longer fragments and prioritizing speed by omitting the DNA repair and adding the adapters through a transposase [74]. In our experience, the ligation-based approach results in substantially larger amounts of data, and filtering short reads allows us to improve the read length distribution. Although the successive sequencing of both strands of a DNA molecule is possible with dedicated kits (duplex sequencing), this is usually not beneficial for plant genomics. For ONT sequencing, the ideal is to retain the longest possible DNA throughout sample preparation and library protocol, maximizing read length and fully leveraging the platform’s capacity for very long reads [75].

Post library preparation, the library is loaded onto flow cells, and sequencing is initiated through the MinKNOW control software. Generated data is monitored in real-time and can be analyzed during the sequencing run. The ideal run time depends on genome size, desired coverage, and flow cell health. For small genomes, a few hours are sufficient to achieve high coverage. It is important to monitor the flow cell’s health (available and actively sequencing pores) using MinKNOW to determine the best time to stop a run, as a flow cell can be washed and reused as long as the pores are available. Since sequencing is monitored in real time by MinKNOW, the run can be terminated when the required coverage is reached to save nanopores for the next sequencing project.

### Step 4: Pore-C

**Fig. 3:**
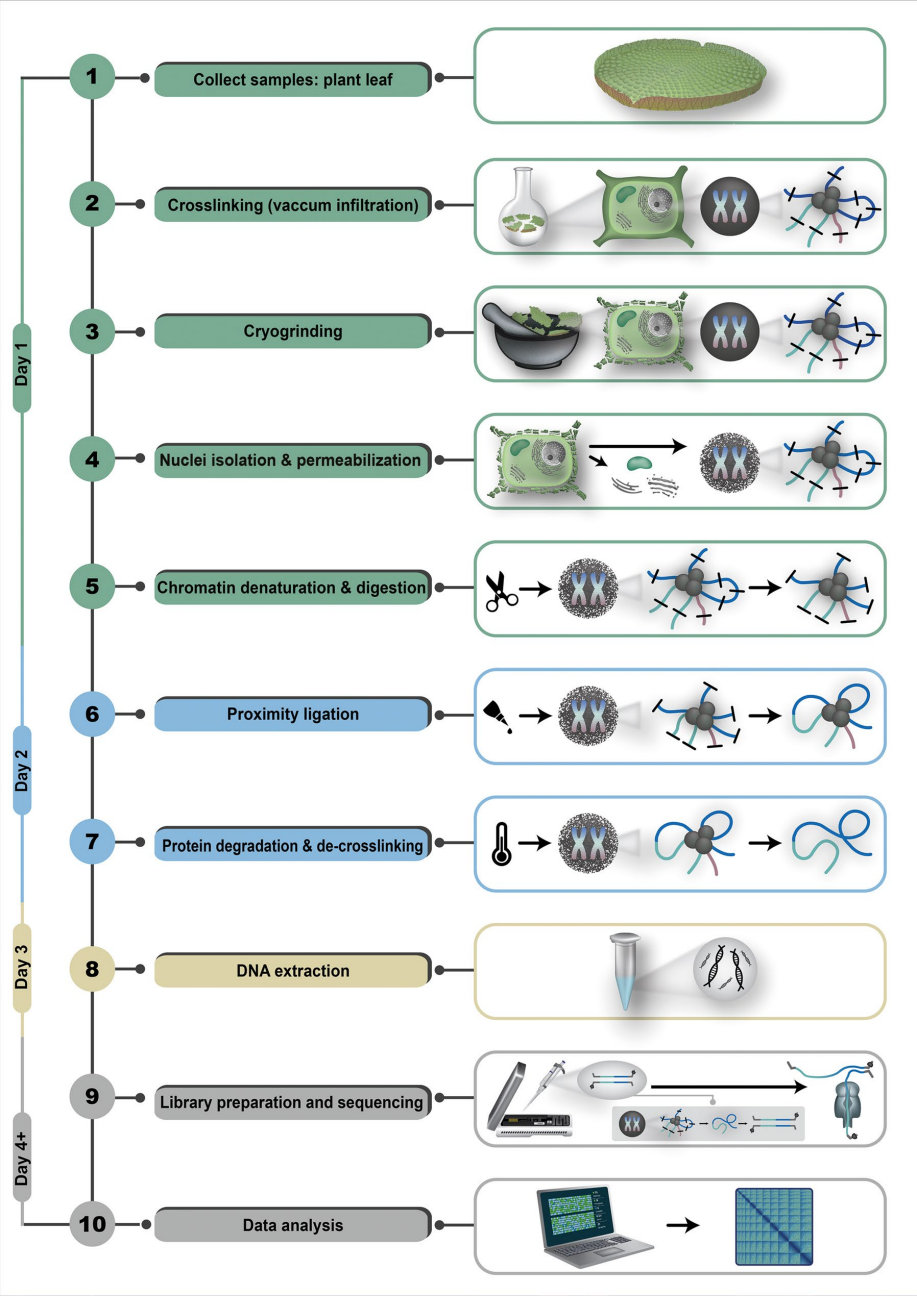
Illustration depicting the typical Pore-C workflow for plant samples. Steps performed on the first day are highlighted in green boxes and include: sample collection, crosslinking, cryogrinding, nuclei isolation, and permeabilization, followed by chromatin denaturation and digestion. Second-day procedures, indicated in blue boxes, involve proximity ligation as well as protein degradation and de-crosslinking. The final wet-lab step performed on the third day is DNA extraction, highlighted in yellow. Subsequent sequencing and data analysis steps are shown in grey. The chromatin is illustrated as three grey circles (proteins) surrounded by colored strands (DNA). The short black lines crossing the illustrated DNA strands mark the restriction enzyme cutting sites

Pore-C is a technique combining chromatin conformation capture (3 C) and nanopore long-read sequencing, similar to how Cifi is the combination of 3 C with PacBio HiFi sequencing [76]. The three-dimensional chromatin structures [77] can be characterized by Pore-C. Compared to previously established 3 C techniques [78], Pore-C does not require DNA amplification steps prior to sequencing and displays a simpler and scalable method for chromatin analysis [79]. The provided protocol [67] represents an adapted end-to-end workflow demonstrated to work well on the water lily *Victoria cruziana*. Completing all steps of the protocol takes three days (Fig. 3). The first step is a chemical crosslinking of DNA and proteins, such as histones, which conserves the spatial arrangement within the nucleus. Compared to animal cell line cultures, plant samples require vacuum pressure for adequate infiltration of formaldehyde during the crosslinking process. After a few infiltration periods, glycine is used to terminate the crosslinking reaction [80]. For plant samples, subsequent cryogrinding in liquid nitrogen is mandatory to disrupt the robust cell wall. For efficient chromatin denaturation, nuclei are isolated and permeabilized using various buffers. These buffers include components such as PVP-40 to remove phenolic compounds [81] for nuclei permeabilization. Subsequently, a low-dose SDS dilution is utilized, combined with low-heat incubation to ensure that crosslinked interactions are maintained as well as the accessibility for restriction enzymes [82]. By default, ONT suggests NlaIII as it is suitable for many species and generates high-density contact outputs with optimal fragment lengths. In theory, an in silico analysis of restriction sites in the genome sequences could reveal areas of reduced cleavage within the genome or repeat-rich genomic regions to determine other restriction enzyme candidates. Obviously, this does not have practical relevance in genome sequencing projects aiming to generate a reference sequence for a new species. During overnight restriction enzyme incubation, clusters of DNA fragments, maintained in proximity through crosslinking, are formed, thereby preserving the native interactions present at the time of crosslinking. Based on the utilized restriction enzyme, either heat or chemical inactivation is performed before starting the proximity ligation. Following DNA ligase administration, the cohesive ends of the crosslinked and digested DNA are ligated into chimeric polymers [78, 82]. After completing ligation, remaining enzymes and proteins are degraded with proteinase K and SDS overnight, resulting in de-crosslinking and the release of chimeric Pore-C polymers as dsDNA [78, 83]. Finally, DNA obtained with the Pore-C protocol can be isolated using a phenol-chloroform extraction with subsequent ethanol precipitation. The phenol-chloroform mixture removes the remaining peptides, whereas ethanol is utilized to purify DNA from retained buffers. Additional supplementation of 5 M NaCl and 3 M sodium acetate, pH 5.5, can enhance DNA precipitation [84]. After extraction, a mixture of chimeric dsDNA molecules that were originally in proximity is obtained. The processed DNA can now be used for size selection, followed by library preparation and sequencing. Protocols for these steps slightly deviate from the standard protocols aiming at the longest possible reads, as chimeric DNA fragments are likely to be substantially shorter [67].

Before library preparation (day 4+), DNA obtained using the Pore-C protocol undergoes an additional size selection step to enrich for fragments greater than 2 kb, which is necessary for optimal Pore-C data generation. This method was originally published by Schalamun & Schwessinger, 2017, and was adapted in the context of Pore-C fragment size selection [85, 86]. The method is based on solid-phase reversible immobilization (SPRI) in which coated paramagnetic beads are utilized to reversibly bind nucleic acids [87], for example, AMPure XP beads (Beckman Coulter). For the size selection process, a custom-prepared buffer with, e.g., AMPure XP beads containing PEG 8000 is utilized. PEG 8000 is responsible for the selective DNA precipitation, whereas smaller fragments are recovered in higher PEG 8000 concentration and larger fragments within a lower concentration [88]. Therefore, an accurate adjustment of PEG 8000 in the custom SPRI solution is crucial to ensure optimal Pore-C fragment selection [89]. However, to maximize the output of chimeric Pore-C fragments, it is recommended to use 0.85X volume of the custom prepared SPRI ratio after appropriate DNA dilution. Prior to library preparation, it is recommended to re-quantify the DNA, as an approximate loss of 50% should be expected [90]. For library preparation, the ligation sequencing kit V14 (SQK-LSK114) can be used with minor variations regarding the DNA repair and end-prep steps [91].

### Step 5: basecalling

Nucleic acids are measured as they pass through a protein nanopore, generating an electrical signal that reflects the chemical composition of the nucleotides within the pore [92]. This signal fluctuates over time as the movement of the nucleic acid alters the nucleotides positioned within the nanopore. Basecalling is the computational step that converts the measured electrical signals into nucleotide sequences that can be used in further analysis. Dorado is the latest basecalling software package from ONT, built on highly optimized deep neural networks that feature both simplex and duplex basecalling modes [93]. Specific models need to be used depending on the flow cell type and sequencing chemistry (example commands in Additional file 1, Step 5). The input format is POD5 and the output format is FASTQ. Dorado provides modification detection capabilities that are valuable for plant epigenomics research since it can detect nucleotide modifications like 5-methylcytosine (5mC), 5-hydroxymethylcytosine (5hmC), and N^6^-methyladenosine (6 mA) directly from raw nanopore signals [94]. Since the technology is capable of measuring any modification, it is also possible to develop customized models to detect other DNA modifications. Plant genome studies have demonstrated that sequence reads produced by Dorado basecalling support high-quality chromosome-scale assemblies and precise DNA methylation analysis [33, 95, 96].

However, it is important to note that Dorado’s models require a powerful GPU to run efficiently, which can be a significant challenge for many users who may not have access to high-performance computational resources locally. To address this limitation, groups without the necessary hardware for bioinformatic analyses can access cloud computing resources for the computationally challenging steps of genomic analyses. Resources dedicated to academics are usually more affordable options and sometimes free of charge. For example, at the national level, the German Network for Bioinformatics Infrastructure (de.NBI) offers free access to cloud-based computing resources and training for researchers, facilitating data analysis without the need for local hardware or advanced computer knowledge. ELIXIR [97] provides support across Europe, while CyVerse [98] can be a valuable resource for researchers in the USA. If no such ‘academic resources’ are available, commercial computing services can be used. Users often pay based on their usage and do not book specific hardware. This makes hardware automatically scalable to the requirements.

### Step 6: read correction

Although the accuracy of ONT raw reads has substantially improved to > 99% (Phred score > 20, Q20+) over the last years, further correction can be helpful. Therefore, sequences obtained through basecalling can be corrected prior to assembly. Several assembly tools, such as NextDenovo [99], HiCanu [100], and Hifiasm [101], utilize an all-vs-all read comparison for correction before the actual assembly process. An alternative is HERRO [102], which allows a correction of the reads before an assembly. Notably, HERRO was developed to be haplotype-aware, enabling chromosome-scale assemblies using HERRO-corrected reads without the need for additional data (example commands in Additional file 1, Step 6). A critical factor in this process is the availability of longer reads, exceeding 100 kbp [103]. While HERRO supports both R9.4.1 and R10.4.1 ONT simplex reads, it is recommended to use it exclusively for R10 reads, particularly given that the developers classify their R9 model as experimental.

### Step 7: genome sequence assembly

The reads generated during sequencing typically do not represent an entire native chromosome and are subject to varying degrees of error. To accurately reconstruct the genome sequence, assembler tools require information on how to connect the reads, which is primarily derived from overlaps between them. Consequently, each segment of the genome must be represented multiple times within the reads. Errors at specific positions in the reads may occur in only a small fraction of them and can be corrected during the assembly process. However, haplotype-resolving assembly programs must be carefully calibrated to avoid misinterpreting native differences between haplotypes as sequencing errors.

Typically, these assembly tools integrate sequence alignment and graph theory-based algorithms to assemble the reads into larger units known as contigs (contiguous sequences). Current state-of-the-art long-read assemblers include Shasta [104], NextDenovo [99], Verkko2 [105], and Hifiasm [101]. Shasta is recognized for its rapid processing speed, making it suitable for preliminary assessments during sequencing projects to determine whether the sequencing reads are adequate or if additional data generation is necessary. Hifiasm was initially developed for PacBio HiFi reads, but has been updated to support ONT R10 simplex reads as well since version 0.21.0-r686 [106]. Verkko2 offers the capability to incorporate Pore-C reads, which are utilized exclusively for non-haplotype-aware scaffolding following the primary assembly process. Different assemblers are optimized for different types of data and genome characteristics. Based on our experience with multiple plant genome sequencing projects, Shasta performs well on high-coverage, high-accuracy R10 HERRO-corrected reads, exhibiting extremely fast runtimes. However, it may produce more fragmented assemblies (lower N50 values) for repeat-rich plant genomes. NextDenovo is not yet optimized for high-accuracy R10 reads but excels on R9 data, generating highly contiguous assemblies even with moderate coverage [35, 107]. According to the authors, Verkko2 can generate telomere-to-telomere (T2T) assemblies when combining ONT reads (preferably read lengths > 100 kb) with PacBio HiFi reads in a hybrid approach [105]. However, in our experience, it performs suboptimally on ONT-only moderate coverage datasets. Hifiasm performs well on high-coverage, high-accuracy ONT R10 reads and can generate haplotype-resolved assemblies when phasing information is available, which is particularly beneficial for heterozygous genomes. In absence of phasing data, it still produces highly contiguous primary assemblies. Given these differences, it is advisable to deploy multiple assemblers (example commands in Additional file 1, Step 7) and select the best assembly based on comprehensive quality metrics (discussed in the next section), tailoring the choice to the species and dataset at hand.

The reconstruction of organellar genomes remains technically challenging for the chondrome, due to its structural complexity, extensive repeats, and frequent recombination events [108]. In contrast, the plastome is highly conserved across species and can typically be assembled without major difficulty [109]. Several organelle-aware assembly tools have been developed, many of which rely on the availability of a suitable reference sequence. For plastomes, ptGAUL provides a dedicated long-read–optimized assembler capable of accurately resolving plastid genomes from noisy or heterogeneous datasets [110]. For mitochondrial genomes, PMAT was specifically developed for the *de novo* assembly of plant chondrome sequences using PacBio HiFi data but can also utilize ONT reads [111]. MitoHiFi offers a workflow tailored to PacBio HiFi reads and can assemble chondrome sequences either directly from raw reads or from mitochondrial contigs extracted from whole-genome assemblies [112]. Similarly, GetOrganelle, developed for short-read data, can also reconstruct both plastid and mitochondrial genomes from long read sequencing derived assembly graphs [113].

Post assembly, it is possible to correct sequence errors in the obtained draft assembly using polishing tools like NextPolish2 [114], medaka [115], or Racon [116]. These polishing tools correct any errors that were introduced during the *de novo* assembly process. However, the leading long-read assembler Hifiasm maintains that it does not require further polishing steps.

### Step 8: assembly evaluation

Once an assembly is obtained, it is essential to evaluate its quality to determine if it meets the project requirements. Assembly quality can be assessed across three major dimensions: continuity, completeness, and correctness.

The contig or scaffold N50 is the most commonly used metric for assessing assembly continuity, as it reflects the length distribution of assembled sequences. It is calculated by sorting all contigs or scaffolds in descending order by length and identifying the shortest sequence among those that collectively comprise at least 50% of the total assembly size. A higher N50 length, approaching the expected length of a chromosome, signifies greater assembly continuity. Related metrics such as N90 (using a 90% threshold) can provide further insights into the continuity of the assembly.

For assembly completeness, Benchmarking Universal Single-Copy Orthologs (BUSCO) [117] and LTR Assembly Index (LAI) [118] can be used. BUSCO measures the presence of highly conserved reference orthologous genes in the target genome sequence. It is essential to select a lineage-specific dataset, as more specific datasets encompass more reference genes, allowing for a more granular benchmark. Given that these datasets typically include genes that are present in 90% of the species within the specified lineage, a BUSCO score of 90% indicates that the assembly is likely to be of high completeness. Additionally, the BUSCO report provides information on the number of genes found to be duplicated. Analyzing these duplicated genes can offer insights into the ploidy status of the sequenced material, particularly when the percentage of duplicates approaches the overall percentage of genes identified. However, since BUSCO relies on gene prediction tools, it can overestimate completeness by incorrectly identifying fragmented and duplicated BUSCOs as complete [117], and there is a risk that future assembly and annotation tools might be optimized towards high BUSCO scores. LAI, on the other hand, estimates the assembly completeness by calculating the percentage of intact LTR-retrotransposons (LTR-RT) in the repetitive regions, which are known to be challenging to resolve in an assembly. This is particularly useful in plant genomes, which often contain a high proportion of LTR-RTs, and has gained popularity recently. Additionally, comparing the assembly size to the expected genome size is an important complementary check. If the assembly size is significantly smaller than expected, it may be incomplete, and additional sequencing data might be needed. Conversely, if the assembly size is substantially larger (e.g., ~2x or 4x), this may indicate that the genetic material is polyploid relative to the anticipated genome size. It is also important to consider that the assembly may inadvertently resolve haplotypes (fully or partially) even when such resolution is not desired, resulting in an assembly size that is approximately twice the expected size. In contrast, the assembly may be roughly half the expected size if haplotypes are merged. MGSE (Mapping-based genome size estimation) has been observed to work well for genome size estimation based on long reads, but requires an initial assembly [119].

Correctness assesses base-level accuracy and structural integrity. For phased assemblies, two metrics are particularly relevant: hamming error and switch error. Hamming error is defined as a misplaced allele on a contig and can be estimated as the proportion of haplotype-specific k-mers incorrectly assigned to the wrong haplotype. A switch error is defined as the change from one parental haplotype to another in a contig. Yak [120] is a widely used k-mer based tool that estimates switch error and hamming error for phased assemblies. Reference-free, k-mer–based approaches such as Merqury [121] provide highly accurate estimates of consensus quality (QV scores) by comparing k-mers from high-accuracy short reads to those present in the assembly. Merqury also quantifies phasing accuracy, like switch error and hamming error, when haplotype-resolved assemblies are available. QUAST [122] can further evaluate both base-level and structural correctness, although its accuracy improves when a high-quality, closely related reference genome sequence is provided. For long-read assemblies, Inspector [123] offers a broad assessment of assembly errors at multiple scales, including large rearrangements, InDels, and phasing switches. More recently, CRAQ (Clipping information for Revealing Assembly Quality) [124] introduced a reference-free framework leveraging both short- and long-read alignments to identify single-nucleotide level errors and structural misjoins, with the added capability of guiding contig correction through breakpoint detection.

In summary, evaluating all three aspects, continuity, completeness, and correctness, is essential for interpretation and improving a genome assembly (example commands to run all mentioned tools in Additional file 1, Step 8). We recommend using multiple tools to capture the full picture of assembly quality and make an informed decision.

### Step 9: scaffolding with Pore-C data

Although modern sequencing technologies increasingly enable chromosome-scale assemblies composed of long contigs, achieving this level of continuity is not always feasible. Following assembly, an additional step called scaffolding links contigs into larger sequences known as scaffolds. Contigs in scaffolds are typically interconnected by multiple “N” bases, which denote an unknown quantity and types of nucleotides at those positions. Scaffolding can be reference-assisted, utilizing a reference genome sequence from the same or closely related species, or it can be enhanced by supplementary data, such as chromosome conformation capture data.

In the context of long-read genome sequencing projects, Pore-C data plays a crucial role in scaffolding assemblies to a chromosome-scale resolution if individual contigs do not represent entire chromosomes. Due to their chimeric nature, Pore-C reads consist of multiple (potentially) distant monomeric sequences [78]. However, the likelihood of these disparate sequences being in spatial proximity is increased when they originate from the same chromosome and, consequently, from the same haplotype [125]. This probability is leveraged during scaffolding. The relationships of contigs can be visualized using a contact map, which highlights the co-occurrences of sequences. Furthermore, Pore-C data facilitates the resolution of haplophases, a process known as phasing, which can produce assemblies that more accurately represent the distinct haplotypes. Tools that leverage Pore-C data, such as the CPhasing tool [126], typically generate a contact map, offering a comprehensive overview for assessing the status of scaffolding.

### Step 10: structural annotation

**Fig. 4:**
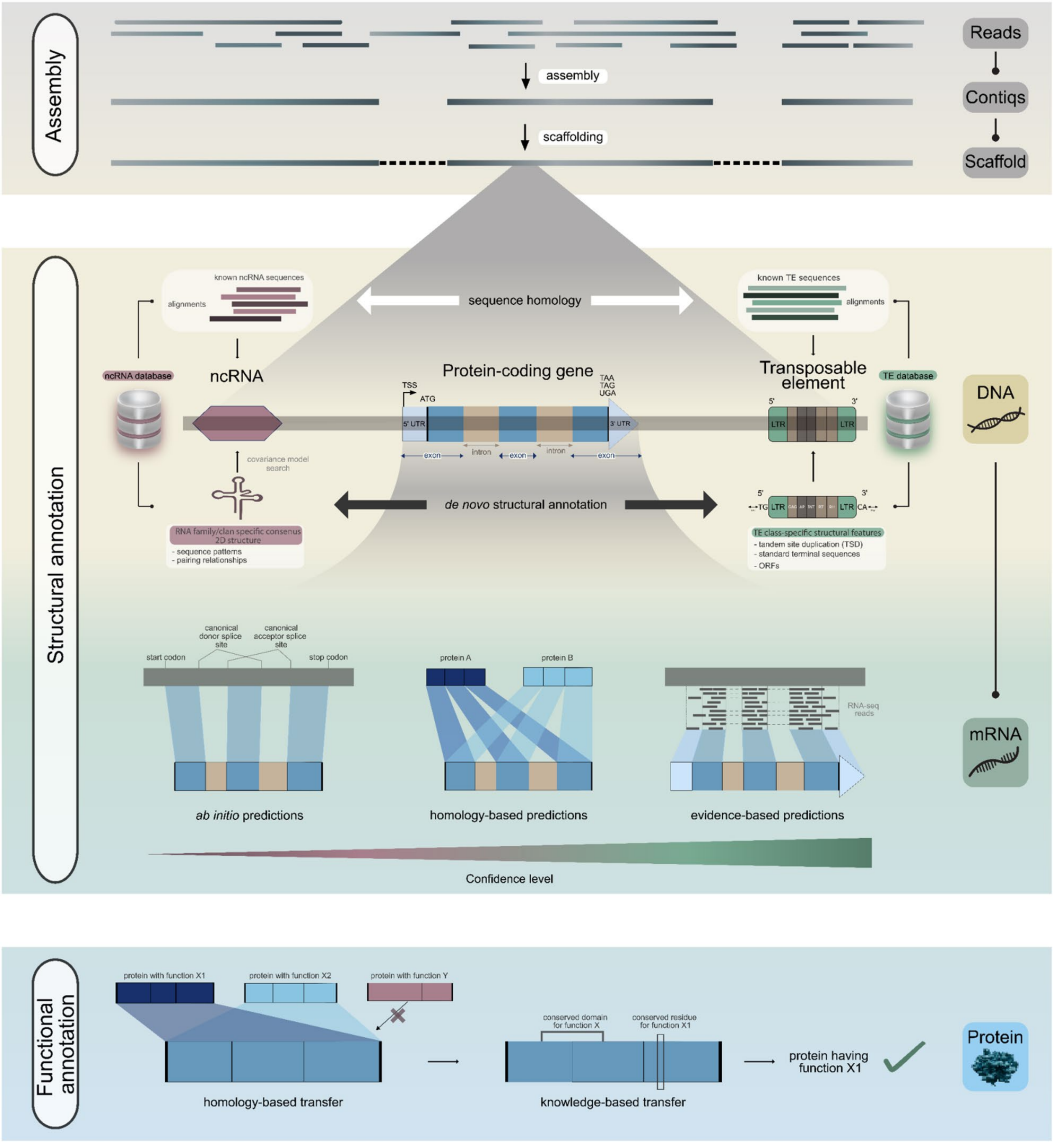
The data analysis steps in a genome sequencing project can be broadly categorized into assembly, structural annotation, and functional annotation. After sequencing and genome assembly, the next step is to efficiently annotate the genome sequence. A genome comprises not only protein-coding genes but also non-coding genes, such as ncRNAs and transposable elements (TEs). Non-coding genes can be annotated either through sequence homology, using known sequences from literature and databases, or more reliably via *de novo* structural annotation, which leverages known sequence patterns and, in the case of ncRNAs, secondary structure and pairing relationships. For protein-coding genes, multiple approaches like ab initio predictions, homology-based predictions, and evidence-based predictions can be combined to generate a high-confidence gene set. Functional annotation can be achieved by transferring annotations from homologous sequences and integrating them with knowledge of conserved domains and residues, resulting in more reliable functional assignments. Abbreviations: ncRNA, non-coding RNA; TE, transposable element; ORF, open reading frame; TSS, transcription start site; UTR, untranslated region; LTR, long terminal repeat

Genome sequence assembly, once the bottleneck of genomics, has now become a routine task. The next and more crucial step is to make sense of these massive strings of A’s, T’s, C’s, and G’s by identifying genes. A gene is defined as a nucleotide sequence that encodes a functional product, which may be either a protein or RNA. This gene prediction process is also referred to as structural annotation. Three major approaches are commonly employed for gene prediction: (a) homology-based predictions using known gene sequences from closely related species, (b) ab initio gene prediction using intrinsic sequence features, and (c) evidence-based predictions incorporating transcriptomic and/or proteomic hints from the same species (Fig. 4). For protein-coding genes, the primary challenge is accurate identification of the exon-intron boundaries, i.e., the splice sites, and the extent of the untranslated regions at the 5’ and 3’ ends. RNA-seq data provide critical evidence for gene prediction, ideally using cDNA-derived sequences from the target species, available from public databases such as NCBI SRA [127] or generated *de novo* if necessary. For homology-based annotation, gene models from closely related species serve as valuable references. GeMoMa [128] utilizes a homology-based approach, integrating MMseqs2 [129] for fast alignments, and optionally incorporating RNA-seq data to improve splice site detection. We recommend selecting high-quality annotations from the closest species available, preferably within the same family, and expanding taxonomic distance only if no closer high-quality references are available. BRAKER [130] is a widely used tool offering flexible combinations of ab initio, homology, and transcriptomics-based evidence. It uses GENEMARK-ETP [131] and AUGUSTUS [132] for gene model training. Similarly, Funannotate [133] integrates multiple tools such as Trimmomatic [134], Trinity [135], PASA [136], HISAT2 [137], and Kallisto [138] for transcriptome processing, followed by AUGUSTUS [132], GlimmerHMM [139], SNAP [140] for prediction, and EvidenceModeler [141] for consensus gene model generation. In our experience, across multiple plant species, BRAKER provides faster runtimes and better performance compared to Funannotate when evaluated using BUSCO scores as a proxy for annotation quality [142].

In exceptional cases where transcriptomic data is unavailable and generating new data is not feasible, multi-species RNA-seq mapping (preferably within the same genus or at least the same family) has been seen to improve annotation quality alongside homology-based predictions [142]. *The ab initio* approach should mainly be reserved for non-model species lacking transcriptomic data and high-quality annotations from closely related taxa [143–145]. Helixer [146] and Tiberius [147] are two modern ab initio gene prediction tools for structural annotation that utilize deep-learning approaches. The most accurate and complete annotations are typically achieved by combining multiple approaches, especially homology and transcriptome-based approaches [33, 142]. For ease and accuracy, we recommend combining GeMoMa and BRAKER predictions, followed by filtering with GeMoMa Annotation Filter (GAF) to obtain a high-confidence gene set (see Additional file 1, Step 10.1.5 for example commands).

Since there is no truly “perfect” genome annotation available and some genes can be species-specific, assessing the validity or near-completeness of a genome annotation is tricky. A simple yet effective strategy is to compare the BUSCO score of the genome assembly with that of the produced annotation, using the same lineage dataset and parameters. A well-annotated genome sequence typically leads to a BUSCO completeness score within ± 2% of the assembly score, indicating that the annotation successfully recovered nearly all genes that BUSCO detected in the assembly. Additionally, visual inspection of predicted gene models using a genome browser like Integrative Genomics Viewer (IGV) [148], ideally along with RNA-seq alignments (in BAM format), provides qualitative validation.

In plants, protein-coding genes often account for only ~ 1–10% of total genome size. The majority of the plant genome consists of non-coding DNA, including transposable elements (TEs), regulatory elements, and various classes of non-coding RNAs (ncRNAs). Accurate identification of these genomic components is equally important. For transposable element annotation, the Extensive *de novo* TE Annotator (EDTA) [149] pipeline provides a comprehensive, automated solution by integrating several high-performing TE annotation tools with appropriate filtering steps. For non-coding RNAs, Infernal [150] offers a sensitive and efficient method for homology-based detection of ncRNAs. Additional specialized tools include tRNAscan-SE [151] for tRNAs, SSU-ALIGN [152] and RNAmmer [153] for rRNA annotation, among others.

For the annotation of organellar genome sequences, the web tool GeSeq employs multiple other tools for structural and functional annotation and can generate a visualization of the annotated organellar genome sequence [154]. Alternatively, chondrome annotation can be conducted with PMGA and plastome annotation with CPGAVAS2, each available as a webtool and for local installation [155, 156].

### Step 11: functional annotation

With the rapid progress in long-read sequencing and the frequent release of new plant genome sequences, understanding the functions of genes as the genetic units becomes the new challenge [157]. The classical approach of knocking out genes and investigating the mutant phenotype is not suitable for all plant species and is not scalable. Therefore, alternative approaches like the inference of gene functions based on orthology are required. The underlying assumption is that orthologs, the same gene in different species, have still maintained the same function since their split in the last common ancestor. A number of tools utilize sequence similarity as a proxy for orthology, as it can be easily measured with tools like BLAST [158] or DIAMOND [159]. Reciprocal best BLAST hits (RBHs) are a popular strategy to improve the accuracy of ortholog identification via local sequence similarity analysis without generating prohibitive computational costs [160]. The identification of conserved domains allows for inferring functional annotation beyond orthologs. An established tool that can integrate protein domain information from different databases is InterProScan5 [161]. When it comes to well-characterized enzymes, detailed knowledge about functionally important amino acid residues, e.g., in the active center, can be utilized [162]. For a general annotation, the annotate function of the Funannotate pipeline [133] offers a comprehensive solution by integrating diverse resources. It can pull and combine functional information from PFAM [163], InterPro [164], EggNog [165], UniProtKB [166], MEROPS [167], CAzyme [168], GO terms [169], and user-provided custom annotations. Additionally, Funannotate can produce NCBI submission-ready files, making it useful for genome projects aiming at public deposition.

### Step 12: data submission

After sequencing and data analysis, the resulting data should be made publicly available to comply with the FAIR data principles [170]. Open data is important to maintain trust in the analysis results by enabling others to reproduce findings and also to enable future data reuse studies [171, 172]. Releasing the electrical signal data would allow future studies to benefit from improvements in basecalling algorithms, which might lead to new discoveries within the same dataset. Additionally, the reads generated through basecalling should be made available to facilitate re-use without computationally expensive basecalling. Typically, this can be accomplished by submitting data to one of the databases of the INSDC, namely ENA [173], SRA [127], or DRA [174]. The basic structure of an INSDC data submission is organized into a BioProject that contains BioSample(s) and sequencing runs. For ONT reads, data of a sequencing run include POD5 (electrical signal data) and FASTQ files (reads). Furthermore, a project may encompass analysis objects or elements that represent genome assemblies and annotation data. Genome assemblies can be submitted as FASTA files. Submission of annotated genome sequences to the INSDC databases necessitates that a locus tag must be registered for the study, and the data is formatted according to the database specifications. However, before formatting, the annotation must be in a valid GFF3 format, free of structural errors. We recommend using AGAT toolkit’s [175] agat_convert_sp_gxf2gxf.pl script to standardize the GFF3 file, followed by agat_sp_fix_features_locations_duplicated.pl to remove features with duplicated locations, which are commonly flagged by ENA and NCBI. Some issues, such as incorrect exons, often require manual curation, and it is advisable to inspect and manually update erroneous gene model(s) before submission. The structural (and functional, if available) annotation results formatted in GFF3 must be integrated with the assembly FASTA into the appropriate submission format, i.e., .tbl file for NCBI submission or an EMBL flat file format for ENA. This can be accomplished using the Genome Annotation generator (GAG) [176] for NCBI or the EMBLmyGFF3 tool [177] for ENA. As INSDC members synchronize their data daily, submissions to any one database are accessible through all three. For NCBI submission, the resulting .tbl needs to be converted to .ASN format using table2asn, which also outputs validation and discrepancy reports, which should be thoroughly reviewed and corrected. Similarly, for EMBL submission, the validation can be done using Webin-CLI. Final submissions are made via the NCBI submission portal [178] (web interface) or through Webin-CLI (command-line interface) [179] for ENA. Once submitted, the assemblies undergo automated validation checks, and if no significant issues are found, the submission is assigned an accession number and becomes publicly accessible.

## Summary

Here we provide a practical and comprehensive guide to plant genomics, covering the entire workflow from project planning to data submission. We begin by outlining strategies for evaluating existing genomic data and estimating required resources, ensuring project efficiency. We then detail optimized protocols for high-quality DNA extraction, adapted to long-read sequencing technologies, followed by best practices for library preparation. The subsequent sections address genome assembly and structural and functional annotations to transform sequence data into relevant biological information. Finally, we address data submission procedures and recommend best practices to ensure robust and reproducible plant genomics research. To facilitate practical application, we provide a comprehensive ‘cheat sheet’ with specific command-line interface (CLI) commands in the Additional file 1 [22] covering all key steps of the workflow discussed in this review. Given that the majority of land plant species remain unsequenced, this field is expected to remain dynamic and expansive. This review offers a timely and practical framework for researchers to initiate and execute long-read plant genome sequencing projects effectively, laying a strong foundation for future functional and evolutionary studies, as well as practical applications in agriculture, biotechnology, and beyond.

## Supplementary Information

Supplementary Material 1.

## Data Availability

The hands-on commands accompanying this article are openly available in the GitHub repository https://github.com/bpucker/PlantGenomicsGuide and in the Codeberg repository https://codeberg.org/PuckerLab/PlantGenomicsGuide. The hands-on guide is also provided as Additional file 1.

## References

[1] The Arabidopsis Genome Initiative. Analysis of the genome sequence of the flowering plant Arabidopsis Thaliana. Nat Nat Publishing Group. 2000;408:796–815. 10.1038/35048692. 10.1038/35048692 11130711

[2] Michael TP, Jupe F, Bemm F, Motley ST, Sandoval JP, Lanz C, et al. High contiguity Arabidopsis Thaliana genome assembly with a single nanopore flow cell. Nat Commun Nat Publishing Group. 2018;9:541. 10.1038/s41467-018-03016-2. 10.1038/s41467-018-03016-2 PMC5803254 29416032

[3] Pucker B, Kleinbölting N, Weisshaar B. Large scale genomic rearrangements in selected Arabidopsis Thaliana T-DNA lines are caused by T-DNA insertion mutagenesis. BMC Genomics. 2021;22:599. 10.1186/s12864-021-07877-8. 34362298 10.1186/s12864-021-07877-8 PMC8348815

[4] Pucker B, Irisarri I, de Vries J, Xu B. Plant genome sequence assembly in the era of long reads: Progress, challenges and future directions. Quant Plant Biol. 2022;3:e5. 10.1017/qpb.2021.18. 37077982 10.1017/qpb.2021.18 PMC10095996

[5] Marks RA, Hotaling S, Frandsen PB, VanBuren R. Representation and participation across 20 years of plant genome sequencing. Nat Plants Nat Publishing Group. 2021;7:1571–8. 10.1038/s41477-021-01031-8. 10.1038/s41477-021-01031-8 PMC8677620 34845350

[6] Schwacke R, Bolger ME, Usadel B. PubPlant -a continuously updated online resource for sequenced and published plant genomes. Front Plant Sci Front. 2025;16. 10.3389/fpls.2025.1603547. 10.3389/fpls.2025.1603547 PMC12235464 40630734

[7] Saha D, Panda AK, Datta S. Critical considerations and computational tools in plant genome editing. Heliyon. 2025;11:e41135. 10.1016/j.heliyon.2024.e41135. 39807514 10.1016/j.heliyon.2024.e41135 PMC11728886

[8] Hyun JC, Monk JM, Palsson BO. Comparative pangenomics: analysis of 12 microbial pathogen pangenomes reveals conserved global structures of genetic and functional diversity. BMC Genomics. 2022;23:7. 10.1186/s12864-021-08223-8. 34983386 10.1186/s12864-021-08223-8 PMC8725406

[9] Liao W-W, Asri M, Ebler J, Doerr D, Haukness M, Hickey G, et al. A draft human pangenome reference. Nat Nat Publishing Group. 2023;617:312–24. 10.1038/s41586-023-05896-x. 10.1038/s41586-023-05896-x PMC10172123 37165242

[10] Meng Q, Xie P, Xu Z, Tang J, Hui L, Gu J, et al. Pangenome analysis reveals yield- and fiber-related diversity and interspecific gene flow in Gossypium Barbadense L. Nat Commun Nat Publishing Group. 2025;16:4995. 10.1038/s41467-025-60254-x. 10.1038/s41467-025-60254-x PMC12122945 40442108

[11] GBIF Secretariat. GBIF Backbone Taxonomy. 2023. 10.15468/39omei

[12] Bachman SP, Brown MJM, Leão TCC, Nic Lughadha E, Walker BE. Extinction risk predictions for the world’s flowering plants to support their conservation. New Phytol. 2024;242:797–808. 10.1111/nph.19592. 38437880 10.1111/nph.19592

[13] The Angiosperm Phylogeny Group, Chase MW, Christenhusz MJM, Fay MF, Byng JW, Judd WS, et al. An update of the angiosperm phylogeny group classification for the orders and families of flowering plants: APG IV. Bot J Linn Soc. 2016;181:1–20. 10.1111/boj.12385.

[14] Janssens SB, Couvreur TLP, Mertens A, Dauby G, Dagallier L-PMJ, Abeele SV, et al. A large-scale species level dated angiosperm phylogeny for evolutionary and ecological analyses. Biodivers Data J Pensoft Publishers. 2020;8:e39677. 10.3897/BDJ.8.e39677. 10.3897/BDJ.8.e39677 PMC6987248 32015666

[15] McTavish EJ, Hinchliff CE, Allman JF, Brown JW, Cranston KA, Holder MT, et al. Phylesystem: a git-based data store for community-curated phylogenetic estimates. Bioinformatics. 2015;31:2794–800. 10.1093/bioinformatics/btv276. 25940563 10.1093/bioinformatics/btv276 PMC4547614

[16] Grotewold E, Chappell J, Kellogg EA. Plant Genes, genomes and genetics. John Wiley & Sons, Ltd; 2015. 10.1002/9781118539385.fmatter.

[17] Brooks EG, Elorriaga E, Liu Y, Duduit JR, Yuan G, Tsai C-J, et al. Plant promoters and terminators for High-Precision bioengineering. BioDesign Res Am Association Advancement Sci. 2023;5:0013. 10.34133/bdr.0013. 10.34133/bdr.0013 PMC10328392 37849460

[18] Liang C, Mao L, Ware D, Stein L. Evidence-based gene predictions in plant genomes. Genome Res. 2009;19:1912–23. 10.1101/gr.088997.108. 19541913 10.1101/gr.088997.108 PMC2765265

[19] Srivastava AK, Lu Y, Zinta G, Lang Z, Zhu J-K. UTR-Dependent control of gene expression in plants. Trends Plant Sci. 2018;23:248–59. 10.1016/j.tplants.2017.11.003. 29223924 10.1016/j.tplants.2017.11.003 PMC5828884

[20] Hardy EC, Balcerowicz M. Untranslated yet indispensable—UTRs act as key regulators in the environmental control of gene expression. J Exp Bot. 2024;75:4314–31. 10.1093/jxb/erae073. 38394144 10.1093/jxb/erae073 PMC11263492

[21] Lu D, Liu C, Ji W, Xia R, Li S, Liu Y, et al. Nanopore ultra-long sequencing and adaptive sampling spur plant complete telomere-to-telomere genome assembly. Mol Plant. 2024;17:1773–86. 10.1016/j.molp.2024.10.008. 39420560 10.1016/j.molp.2024.10.008

[22] de Oliveira JAVS, Choudhary N, Meckoni SN, Pucker B. Plant Genomics Guide. 2025. https://github.com/bpucker/PlantGenomicsGuide. Accessed 14 Aug 2025. cited 2025 Aug 14.

[23] Chen G, Sun W. The role of botanical gardens in scientific research, conservation, and citizen science. Plant Divers. 2018;40:181–8. 10.1016/j.pld.2018.07.006. 30740563 10.1016/j.pld.2018.07.006 PMC6137266

[24] Aleza P, Juárez J, Hernández M, Pina JA, Ollitrault P, Navarro L. Recovery and characterization of a citrus clementinaHort. Ex Tan. Clemenules haploid plant selected to Establish the reference whole citrus genome sequence. BMC Plant Biol. 2009;9:110. 10.1186/1471-2229-9-110. 19698121 10.1186/1471-2229-9-110 PMC2747335

[25] Hirsch CN, Hirsch CD, Brohammer AB, Bowman MJ, Soifer I, Barad O, et al. Draft assembly of elite inbred line PH207 provides insights into genomic and transcriptome diversity in maize. Plant Cell. 2016;28:2700–14. 10.1105/tpc.16.00353. 27803309 10.1105/tpc.16.00353 PMC5155341

[26] Schwartz JC, Farrell CP, Freimanis G, Sewell AK, Phillips JD, Hammond JA. A genome assembly and transcriptome atlas of the inbred Babraham pig to illuminate Porcine Immunogenetic variation. Immunogenetics. 2024;76:361–80. 10.1007/s00251-024-01355-7. 39294478 10.1007/s00251-024-01355-7 PMC11496355

[27] Wang B, Jiao Y, Chougule K, Olson A, Huang J, Llaca V, et al. Pan-genome analysis in sorghum highlights the extent of genomic variation and sugarcane aphid resistance genes. BioRxiv. 2021. 10.1101/2021.01.03.424980. p. 2021.01.03.424980.35018379

[28] Yang N, Liu J, Gao Q, Gui S, Chen L, Yang L, et al. Genome assembly of a tropical maize inbred line provides insights into structural variation and crop improvement. Nat Genet Nat Publishing Group. 2019;51:1052–9. 10.1038/s41588-019-0427-6. 10.1038/s41588-019-0427-6 31152161

[29] Ma H, Liu Y, Liu D, Sun W, Liu X, Wan Y, et al. Chromosome-level genome assembly and population genetic analysis of a critically endangered rhododendron provide insights into its conservation. Plant J. 2021;107:1533–45. 10.1111/tpj.15399. 34189793 10.1111/tpj.15399

[30] Pavese V, Cavalet-Giorsa E, Barchi L, Acquadro A, Torello Marinoni D, Portis E et al. Whole-genome assembly of Corylus Avellana Cv Tonda gentile Delle Langhe using linked-reads (10X Genomics). G3 genesgenomesgenetics. 2021;11:jkab152. 10.1093/g3journal/jkab152 10.1093/g3journal/jkab152 PMC8495946 33964151

[31] Ben Romdhane W, Ben Saad R, Guiderdoni E, Ali AA, mohamed, Tarroum M, Al-Doss A, et al. De novo, high-quality assembly and annotation of the halophyte grass aeluropus littoralis draft genome and identification of A20/AN1 zinc finger protein family. BMC Plant Biol. 2025;25:556. 10.1186/s12870-025-06610-x. 40295936 10.1186/s12870-025-06610-x PMC12039208

[32] Liu H, Wei J, Yang T, Mu W, Song B, Yang T, et al. Molecular digitization of a botanical garden: high-depth whole-genome sequencing of 689 vascular plant species from the Ruili botanical garden. GigaScience. 2019;8:giz007. 10.1093/gigascience/giz007. 30689836 10.1093/gigascience/giz007 PMC6441391

[33] Nowak MS, Harder B, Meckoni SN, Friedhoff R, Wolff K, Pucker B. Genome sequence and RNA-seq analysis reveal genetic basis of flower coloration in the giant water Lily Victoria Cruziana. bioRxiv; 2024. p. 2024.06.15.599162. 10.1101/2024.06.15.599162

[34] Recinos MFM, Winnier S, Lagerhausen K, Ajayi B, Wolff K, Friedhoff R et al. Cacao genome sequence reveals insights into the flavonoid biosynthesis. bioRxiv; 2024. p. 2024.11.23.624982. 10.1101/2024.11.23.624982

[35] Hakim SE, Choudhary N, Malhotra K, Peng J, Bültemeier A, Arafa A, et al. Phylogenomics and metabolic engineering reveal a conserved gene cluster in Solanaceae plants for withanolide biosynthesis. Nat Commun Nat Publishing Group. 2025;16:6367. 10.1038/s41467-025-61686-1. 10.1038/s41467-025-61686-1 PMC12246201 40640164

[36] Li B, Dewey CN. RSEM: accurate transcript quantification from RNA-Seq data with or without a reference genome. BMC Bioinformatics. 2011;12:323. 10.1186/1471-2105-12-323. 21816040 10.1186/1471-2105-12-323 PMC3163565

[37] Li Z, Zhang Z, Yan P, Huang S, Fei Z, Lin K. RNA-Seq improves annotation of protein-coding genes in the cucumber genome. BMC Genomics. 2011;12:540. 10.1186/1471-2164-12-540. 22047402 10.1186/1471-2164-12-540 PMC3219749

[38] Arita M, Karsch-Mizrachi I, Cochrane G, on behalf of the International Nucleotide Sequence Database Collaboration. The international nucleotide sequence database collaboration. Nucleic Acids Res. 2021;49:D121–4. 10.1093/nar/gkaa967. 33166387 10.1093/nar/gkaa967 PMC7778961

[39] Goodstein DM, Shu S, Howson R, Neupane R, Hayes RD, Fazo J, et al. Phytozome: a comparative platform for green plant genomics. Nucleic Acids Res. 2012;40:D1178–86. 10.1093/nar/gkr944. 22110026 10.1093/nar/gkr944 PMC3245001

[40] CNCB-NGDC Members and Partners. Database Resources of the National Genomics Data Center. China National center for bioinformation in 2025. Nucleic Acids Res. 2025;53:D30. 10.1093/nar/gkae978. 39530327 10.1093/nar/gkae978 PMC11701749

[41] Wani ZA, Bhat A, Figshare. A One-Stop shop for research data management with diverse features and services. J Inf Knowl. 2022;59:391–7. 10.17821/srels/2022/v59i6/170789.

[42] Vision T. The Dryad Digital Repository: Published evolutionary data as part of the greater data ecosystem. Nat Preced Nat Publishing Group. 2010;1. 10.1038/npre.2010.4595.1.

[43] Arend D, Lange M, Chen J, Colmsee C, Flemming S, Hecht D, et al. e!DAL - a framework to store, share and publish research data. BMC Bioinformatics. 2014;15:214. 10.1186/1471-2105-15-214. 24958009 10.1186/1471-2105-15-214 PMC4080583

[44] Arend D, Junker A, Scholz U, Schüler D, Wylie J, Lange M. PGP repository: a plant phenomics and genomics data publication infrastructure. Database. 2016;2016:baw033. 10.1093/database/baw033. 27087305 10.1093/database/baw033 PMC4834206

[45] Pellicer J, Leitch IJ. The plant DNA C-values database (release 7.1): an updated online repository of plant genome size data for comparative studies. New Phytol. 2020;226:301–5. 10.1111/nph.16261. 31608445 10.1111/nph.16261

[46] Dohm JC, Minoche AE, Holtgräwe D, Capella-Gutiérrez S, Zakrzewski F, Tafer H, et al. The genome of the recently domesticated crop plant sugar beet (Beta vulgaris). Nat Nat Publishing Group. 2014;505:546–9. 10.1038/nature12817. 10.1038/nature12817 24352233

[47] Shi X, Cao S, Wang X, Huang S, Wang Y, Liu Z, et al. The complete reference genome for grapevine (Vitis vinifera L.) genetics and breeding. Hortic Res. 2023;10:uhad061. 10.1093/hr/uhad061. 37213686 10.1093/hr/uhad061 PMC10199708

[48] Heslop-Harrison JS (Pat), Schwarzacher T, Liu Q, editors. Polyploidy: its consequences and enabling role in plant diversification and evolution. Ann Bot. 2023;131:1–10. 10.1093/aob/mcac132 10.1093/aob/mcac132 PMC9904344 36282971

[49] Duan H, Jones AW, Hewitt T, Mackenzie A, Hu Y, Sharp A, et al. Physical separation of haplotypes in dikaryons allows benchmarking of phasing accuracy in nanopore and HiFi assemblies with Hi-C data. Genome Biol. 2022;23:84. 10.1186/s13059-022-02658-2. 35337367 10.1186/s13059-022-02658-2 PMC8957140

[50] Sarashetti P, Lipovac J, Tomas F, Šikić M, Liu J. Evaluating data requirements for high-quality haplotype-resolved genomes for creating robust pangenome references. Genome Biol. 2024;25:312. 10.1186/s13059-024-03452-y. 39696427 10.1186/s13059-024-03452-y PMC11658127

[51] Pellicer J, Kelly LJ, Magdalena C, Leitch IJ. Insights into the dynamics of genome size and chromosome evolution in the early diverging angiosperm lineage nymphaeales (water lilies). Genome. NRC Res Press. 2013;56:437–49. 10.1139/gen-2013-0039. 10.1139/gen-2013-0039 24168627

[52] Marçais G, Kingsford C. A fast, lock-free approach for efficient parallel counting of occurrences of k-mers. Bioinformatics. 2011;27:764–70. 10.1093/bioinformatics/btr011. 21217122 10.1093/bioinformatics/btr011 PMC3051319

[53] Kokot M, Długosz M, Deorowicz S. KMC 3: counting and manipulating k-mer statistics. Bioinformatics. 2017;33:2759–61. 10.1093/bioinformatics/btx304. 28472236 10.1093/bioinformatics/btx304

[54] Ranallo-Benavidez TR, Jaron KS, Schatz MC. GenomeScope 2.0 and smudgeplot for reference-free profiling of polyploid genomes. Nat Commun Nat Publishing Group. 2020;11:1432. 10.1038/s41467-020-14998-3. 10.1038/s41467-020-14998-3 PMC7080791 32188846

[55] Espinosa E, Bautista R, Larrosa R, Plata O. Advancements in long-read genome sequencing technologies and algorithms. Genomics. 2024;116:110842. 10.1016/j.ygeno.2024.110842. 38608738 10.1016/j.ygeno.2024.110842

[56] Russo A, Mayjonade B, Frei D, Potente G, Kellenberger RT, Frachon L, et al. Low-Input High-Molecular-Weight DNA extraction for Long-Read sequencing from plants of diverse families. Front Plant Sci Front. 2022;13. 10.3389/fpls.2022.883897. 10.3389/fpls.2022.883897 PMC9161206 35665166

[57] Friar EA. Isolation of DNA from plants with large amounts of secondary metabolites. Methods Enzymol. Academic; 2005. p. 1. 10.1016/S0076-6879(05)95001-5. 10.1016/S0076-6879(05)95001-5 15865957

[58] Rosso MG, Li Y, Strizhov N, Reiss B, Dekker K, Weisshaar B. An Arabidopsis Thaliana T-DNA mutagenized population (GABI-Kat) for flanking sequence tag-based reverse genetics. Plant Mol Biol. 2003;53:247–59. 10.1023/B:PLAN.0000009297.37235.4a. 14756321 10.1023/B:PLAN.0000009297.37235.4a

[59] Doyle JJ, Doyle JL. Isolation of plant DNA from fresh tissue. Focus. 1990;12:13–5.

[60] Siadjeu C, Pucker B, Viehöver P, Albach DC, Weisshaar B. High contiguity de Novo genome sequence assembly of trifoliate Yam (Dioscorea dumetorum) using long read Sequencing. Genes. 2020;11:274. 10.3390/genes11030274. 10.3390/genes11030274 PMC7140821 32143301

[61] Pucker P B. Plant DNA extraction and Preparation for ONT sequencing. Protocols Io. 2020. 10.17504/protocols.io.bcvyiw7w.

[62] Zhang Y, Zhang Y, Burke JM, Gleitsman K, Friedrich SM, Liu KJ, et al. A simple thermoplastic substrate containing hierarchical silica lamellae for high molecular weight DNA extraction. Adv Mater Deerfield Beach Fla. 2016;28:10630–6. 10.1002/adma.201603738. 10.1002/adma.201603738 PMC5234087 27862402

[63] Butto T, Mungikar K, Baumann P, Winter J, Lutz B, Gerber S. Nuclei on the rise: when Nuclei-Based methods Meet Next-Generation sequencing. Cells Multidisciplinary Digit Publishing Inst. 2023;12:1051. 10.3390/cells12071051. 10.3390/cells12071051 PMC10093037 37048124

[64] Kang M, Chanderbali A, Lee S, Soltis DE, Soltis PS, Kim S. High-molecular-weight DNA extraction for long-read sequencing of plant genomes: An optimization of standard methods. Appl Plant Sci. John Wiley & Sons, Ltd; 2023;11:e11528. 10.1002/aps3.11528 10.1002/aps3.11528 PMC10278927 37342161

[65] Ling G, Waxman DJ. Isolation of nuclei for use in Genome-wide DNase hypersensitivity assays to probe chromatin structure. Methods Mol Biol Clifton NJ. 2013;977:13–9. 10.1007/978-1-62703-284-1_2. 10.1007/978-1-62703-284-1_2 PMC3815455 23436350

[66] Zerpa-Catanho D, Zhang X, Song J, Hernandez AG, Ming R. Ultra-long DNA molecule isolation from plant nuclei for ultra-long read genome sequencing. STAR Protoc. 2021;2:100343. 10.1016/j.xpro.2021.100343. 33665623 10.1016/j.xpro.2021.100343 PMC7902544

[67] Nowak MS, Pucker B. Pore-C Protocol for Plant Samples. protocols.io. 2025; 10.17504/protocols.io.rm7vz9mmrgx1/v1

[68] Zhang M, Zhang Y, Scheuring CF, Wu C-C, Dong JJ, Zhang H-B. Preparation of megabase-sized DNA from a variety of organisms using the nuclei method for advanced genomics research. Nat Protoc Nat Publishing Group. 2012;7:467–78. 10.1038/nprot.2011.455. 10.1038/nprot.2011.455 22343429

[69] Workman R, Fedak R, Kilburn D, Hao S, Liu K, Timp W. High molecular weight DNA extraction from recalcitrant plant species for third generation sequencing. Protocols Io. 2019. 10.17504/protocols.io.4vbgw2n.

[70] Li Z, Parris S, Saski CA. A simple plant high-molecular-weight DNA extraction method suitable for single-molecule technologies. Plant Methods. 2020;16:38. 10.1186/s13007-020-00579-4. 32190102 10.1186/s13007-020-00579-4 PMC7071634

[71] Mayjonade B, Gouzy,Jérôme D et al. Cécile, Pouilly, Nicolas, Marande, William, Callot, Caroline,. Extraction of High-Molecular-Weight Genomic DNA for Long-Read Sequencing of Single Molecules. BioTechniques. Taylor & Francis; 2016;61:203–5. 10.2144/000114460. 10.2144/000114460 27712583

[72] Jones A, Torkel C, Stanley D, Nasim J, Borevitz J, Schwessinger B. Scalable high-molecular weight DNA extraction for long-read sequencing. Protocols Io. 2020. 10.17504/protocols.io.bnjhmcj6. 10.1371/journal.pone.0253830 PMC8282028 34264958

[73] Jones A, Torkel C, Stanley D, Nasim J, Borevitz J, Schwessinger B. High-molecular weight DNA extraction, clean-up and size selection for long-read sequencing. PLOS ONE Public Libr Sci. 2021;16:e0253830. 10.1371/journal.pone.0253830. 10.1371/journal.pone.0253830 PMC8282028 34264958

[74] Sauvage T, Cormier A, Delphine P. A comparison of Oxford nanopore library strategies for bacterial genomics. BMC Genomics. 2023;24:627. 10.1186/s12864-023-09729-z. 37864145 10.1186/s12864-023-09729-z PMC10589936

[75] Jain M, Koren S, Miga KH, Quick J, Rand AC, Sasani TA, et al. Nanopore sequencing and assembly of a human genome with ultra-long reads. Nat Biotechnol Nat Publishing Group. 2018;36:338–45. 10.1038/nbt.4060. 10.1038/nbt.4060 PMC5889714 29431738

[76] McGinty SP, Kaya G, Sim SB, Makunin A, Corpuz RL, Quail MA, et al. CiFi: Accurate long-read chromosome conformation capture with low-input requirements. bioRxiv; 2025. p. 2025.01.31.635566. 10.1038/s41467-025-66918-y. cited 2025 Dec 9. 10.1038/s41467-025-66918-y PMC12780124 41360792

[77] Dekker J, Rippe K, Dekker M, Kleckner N. Capturing chromosome conformation. Sci Am Association Advancement Sci. 2002;295:1306–11. 10.1126/science.1067799. 10.1126/science.1067799 11847345

[78] Deshpande AS, Ulahannan N, Pendleton M, Dai X, Ly L, Behr JM, et al. Identifying synergistic high-order 3D chromatin conformations from genome-scale nanopore concatemer sequencing. Nat Biotechnol Nat Publishing Group. 2022;40:1488–99. 10.1038/s41587-022-01289-z. 10.1038/s41587-022-01289-z 35637420

[79] Sati S, Cavalli G. Chromosome conformation capture technologies and their impact in Understanding genome function. Chromosoma. 2017;126:33–44. 10.1007/s00412-016-0593-6. 27130552 10.1007/s00412-016-0593-6

[80] Hoffman EA, Frey BL, Smith LM, Auble DT. Formaldehyde crosslinking: A tool for the study of chromatin complexes. J Biol Chem. 2015;290:26404–11. 10.1074/jbc.R115.651679. 26354429 10.1074/jbc.R115.651679 PMC4646298

[81] Sikorskaite S, Rajamäki M-L, Baniulis D, Stanys V, Valkonen JP, Protocol. Optimised methodology for isolation of nuclei from leaves of species in the Solanaceae and rosaceae families. Plant Methods. 2013;9:31. 10.1186/1746-4811-9-31. 23886449 10.1186/1746-4811-9-31 PMC3728069

[82] Belton J-M, McCord RP, Gibcus JH, Naumova N, Zhan Y, Dekker J. Hi–C: A comprehensive technique to capture the conformation of genomes. Methods. 2012;58:268–76. 10.1016/j.ymeth.2012.05.001. 22652625 10.1016/j.ymeth.2012.05.001 PMC3874846

[83] Weber K, Kuter DJ. Reversible denaturation of enzymes by sodium Dodecyl sulfate. J Biol Chem. 1971;246:4504–9. 10.1016/S0021-9258(18)62040-X. 5106387

[84] Zhong J-Y, Niu L, Lin Z-B, Bai X, Chen Y, Luo F, et al. High-throughput Pore-C reveals the single-allele topology and cell type-specificity of 3D genome folding. Nat Commun Nat Publishing Group. 2023;14:1250. 10.1038/s41467-023-36899-x. 10.1038/s41467-023-36899-x PMC9988853 36878904

[85] Schalamun M, Schwessinger B. DNA size selection (> 1 kb) and clean up using an optimized SPRI beads mixture. Protocols Io. 2017. 10.17504/protocols.io.idmca46.

[86] Schalamun M, Nagar R, Kainer D, Beavan E, Eccles D, Rathjen JP, et al. Harnessing the minion: an example of how to Establish long-read sequencing in a laboratory using challenging plant tissue from Eucalyptus pauciflora. Mol Ecol Resour. 2018;19:77–89. 10.1111/1755-0998.12938. 30118581 10.1111/1755-0998.12938 PMC7380007

[87] DeAngelis MM, Wang DG, Hawkins TL. Solid-phase reversible immobilization for the isolation of PCR products. Nucleic Acids Res. 1995;23:4742–3. 10.1093/nar/23.22.4742. 8524672 10.1093/nar/23.22.4742 PMC307455

[88] Lis JT, Schleif R. Size fractionation of double-stranded DNA by precipitation with polyethylene glycol. Nucleic Acids Res. 1975;2:383–90. 10.1093/nar/2.3.383. 236548 10.1093/nar/2.3.383 PMC342844

[89] He Z, Zhu Y, Gu H. A new method for the determination of critical polyethylene glycol concentration for selective precipitation of DNA fragments. Appl Microbiol Biotechnol. 2013;97:9175–83. 10.1007/s00253-013-5195-0. 23982329 10.1007/s00253-013-5195-0

[90] Restriction enzyme Pore-C info sheet. Oxf. Nanopore Technol. 2019. https://nanoporetech.com/document/restriction-enzyme-pore-c. Accessed 23 July 2025. cited 2025 July 23.

[91] Ligation sequencing DNA V14 (SQK-LSK114). Oxf. Nanopore Technol. 2022. https://nanoporetech.com/document/genomic-dna-by-ligation-sqk-lsk114. Accessed 23 July 2025. cited 2025 July 23.

[92] Pagès-Gallego M, de Ridder J. Comprehensive benchmark and architectural analysis of deep learning models for nanopore sequencing basecalling. Genome Biol. 2023;24:71. 10.1186/s13059-023-02903-2. 37041647 10.1186/s13059-023-02903-2 PMC10088207

[93] Dorado Documentation. https://dorado-docs.readthedocs.io/en/latest/. Accessed 23 July 2025. cited 2025 July 23.

[94] Dorado. Oxford Nanopore Technologies. https://github.com/nanoporetech/dorado. Accessed 23 July 2025. cited 2025 July 23.

[95] Koren S, Bao Z, Guarracino A, Ou S, Goodwin S, Jenike KM, et al. Gapless assembly of complete human and plant chromosomes using only nanopore sequencing. Genome Res Cold Spring Harbor Lab. 2024;34:1919–30. 10.1101/gr.279334.124. 10.1101/gr.279334.124 PMC11610574 39505490

[96] Krawczyk K, Szablińska-Piernik J, Paukszto Ł, Maździarz M, Sulima P, Przyborowski JA, et al. Chromosome-scale telomere to telomere genome assembly of common crystalwort (Riccia Sorocarpa Bisch). Sci Data. 2025;12:77. 10.1038/s41597-025-04373-6. 39814758 10.1038/s41597-025-04373-6 PMC11735767

[97] ELIXIR. https://elixir-europe.org/. Accessed 23 July 2025. cited 2025 July 23.

[98] CyVerse. https://cyverse.org/. Accessed 23 July 2025. cited 2025 July 23.

[99] Hu J, Wang Z, Sun Z, Hu B, Ayoola AO, Liang F, et al. NextDenovo: an efficient error correction and accurate assembly tool for noisy long reads. Genome Biol. 2024;25:107. 10.1186/s13059-024-03252-4. 38671502 10.1186/s13059-024-03252-4 PMC11046930

[100] Nurk S, Walenz BP, Rhie A, Vollger MR, Logsdon GA, Grothe R, et al. HiCanu: accurate assembly of segmental duplications, satellites, and allelic variants from high-fidelity long reads. Genome Res. 2020;30:1291–305. 10.1101/gr.263566.120. 32801147 10.1101/gr.263566.120 PMC7545148

[101] Cheng H, Concepcion GT, Feng X, Zhang H, Li H. Haplotype-resolved de Novo assembly using phased assembly graphs with hifiasm. Nat Methods Nat Publishing Group. 2021;18:170–5. 10.1038/s41592-020-01056-5. 10.1038/s41592-020-01056-5 PMC7961889 33526886

[102] HERRO. Šikić lab. https://github.com/lbcb-sci/herro. Accessed 23 July 2025. cited 2025 July 23.

[103] Stanojevic D, Lin D, Nurk S, Florez De Sessions P, Sikic M. Telomere-to-Telomere Phased Genome Assembly Using HERRO-Corrected Simplex Nanopore Reads. bioRxiv; 2024. 10.1101/2024.05.18.594796.

[104] Shafin K, Pesout T, Lorig-Roach R, Haukness M, Olsen HE, Bosworth C, et al. Nanopore sequencing and the Shasta toolkit enable efficient de Novo assembly of eleven human genomes. Nat Biotechnol Nat Publishing Group. 2020;38:1044–53. 10.1038/s41587-020-0503-6. 10.1038/s41587-020-0503-6 PMC7483855 32686750

[105] Antipov D, Rautiainen M, Nurk S, Walenz BP, Solar SJ, Phillippy AM, et al. Verkko2: integrating proximity ligation data with long-read de Bruijn graphs for efficient telomere-to-telomere genome assembly, phasing, and scaffolding. bioRxiv; 2024. 10.1101/gr.280383.124. 10.1101/gr.280383.124 PMC12212074 40389285

[106] hifiasm. 2025. 10.1038/s41586-026-10105-6. Accessed 23 July 2025. cited 2025 July 23.

[107] Horz JM, Wolff K, Friedhoff R, Pucker B. Genome sequence of the ornamental plant digitalis purpurea reveals the molecular basis of flower color and morphology variation. bioRxiv; 2024. p. 2024.02.14.580303. 10.1101/2024.02.14.580303 10.1186/s12864-026-12889-3 PMC13134276 42067773

[108] Palmer JD, Herbon LA. Plant mitochondrial DNA evolved rapidly in structure, but slowly in sequence. J Mol Evol. 1988;28:87–97. 10.1007/BF02143500. 3148746 10.1007/BF02143500

[109] Palmer JD, COMPARATIVE, ORGANIZATION OF CHLOROPLAST GENOMES. Annu Rev Genet Annual Reviews. 1985;19:325–54. 10.1146/annurev.ge.19.120185.001545. 10.1146/annurev.ge.19.120185.001545 3936406

[110] Zhou W, Armijos CE, Lee C, Lu R, Wang J, Ruhlman TA, et al. Plastid genome assembly using Long-read data. Mol Ecol Resour. 2023;23:1442–57. 10.1111/1755-0998.13787. 36939021 10.1111/1755-0998.13787 PMC10354735

[111] Bi C, Shen F, Han F, Qu Y, Hou J, Xu K, et al. PMAT: an efficient plant mitogenome assembly toolkit using low-coverage HiFi sequencing data. Hortic Res. 2024;11:uhae023. 10.1093/hr/uhae023. 38469379 10.1093/hr/uhae023 PMC10925850

[112] Uliano-Silva M, Ferreira JGRN, Krasheninnikova K, Blaxter M, Mieszkowska N, Hall N, et al. MitoHiFi: a python pipeline for mitochondrial genome assembly from PacBio high fidelity reads. BMC Bioinformatics. 2023;24:288. 10.1186/s12859-023-05385-y. 37464285 10.1186/s12859-023-05385-y PMC10354987

[113] Jin J-J, Yu W-B, Yang J-B, Song Y, dePamphilis CW, Yi T-S, et al. GetOrganelle: a fast and versatile toolkit for accurate de Novo assembly of organelle genomes. Genome Biol. 2020;21:241. 10.1186/s13059-020-02154-5. 32912315 10.1186/s13059-020-02154-5 PMC7488116

[114] Hu J, Wang Z, Liang F, Liu S-L, Ye K, Wang D-P. NextPolish2: A Repeat-aware Polishing tool for genomes assembled using HiFi long reads. Genomics Proteom Bioinf. 2024;22:qzad009. 10.1093/gpbjnl/qzad009. 10.1093/gpbjnl/qzad009 PMC12016036 38862426

[115] GitHub - nanoporetech/medaka. Sequence correction provided by ONT Research. https://github.com/nanoporetech/medaka. Accessed 11 Dec 2025. cited 2025 Dec 11.

[116] Vaser R, Sović I, Nagarajan N, Šikić M. Fast and accurate de Novo genome assembly from long uncorrected reads. Genome Res Cold Spring Harbor Lab. 2017;27:737–46. 10.1101/gr.214270.116. 10.1101/gr.214270.116 PMC5411768 28100585

[117] Manni M, Berkeley MR, Seppey M, Zdobnov EM. BUSCO: assessing genomic data quality and beyond. Curr Protoc. 2021;1:e323. 10.1002/cpz1.323. 34936221 10.1002/cpz1.323

[118] Ou S, Chen J, Jiang N. Assessing genome assembly quality using the LTR assembly index (LAI). Nucleic Acids Res. 2018;46:e126. 10.1093/nar/gky730. 30107434 10.1093/nar/gky730 PMC6265445

[119] Natarajan S, Gehrke J, Pucker B. Mapping-based genome size Estimation. BMC Genomics. 2025;26:482. 10.1186/s12864-025-11640-8. 40369445 10.1186/s12864-025-11640-8 PMC12079912

[120] Li H. lh3/yak. 2025. https://github.com/lh3/yak. Accessed 9 Dec 2025. cited 2025 Dec 9.

[121] Rhie A, Walenz BP, Koren S, Phillippy AM. Merqury: reference-free quality, completeness, and phasing assessment for genome assemblies. Genome Biol. 2020;21:245. 10.1186/s13059-020-02134-9. 32928274 10.1186/s13059-020-02134-9 PMC7488777

[122] Gurevich A, Saveliev V, Vyahhi N, Tesler G. QUAST: quality assessment tool for genome assemblies. Bioinformatics. 2013;29:1072–5. 10.1093/bioinformatics/btt086. 23422339 10.1093/bioinformatics/btt086 PMC3624806

[123] Chen Y, Zhang Y, Wang AY, Gao M, Chong Z. Accurate long-read de Novo assembly evaluation with inspector. Genome Biol. 2021;22:312. 10.1186/s13059-021-02527-4. 34775997 10.1186/s13059-021-02527-4 PMC8590762

[124] Li K, Xu P, Wang J, Yi X, Jiao Y. Identification of errors in draft genome assemblies at single-nucleotide resolution for quality assessment and improvement. Nat Commun Nat Publishing Group. 2023;14:6556. 10.1038/s41467-023-42336-w. 10.1038/s41467-023-42336-w PMC10582259 37848433

[125] McCord RP, Kaplan N, Giorgetti L. Chromosome conformation capture and beyond: toward an integrative view of chromosome structure and function. Mol Cell. 2020;77:688–708. 10.1016/j.molcel.2019.12.021. 32001106 10.1016/j.molcel.2019.12.021 PMC7134573

[126] CPhasing. 2025. https://github.com/wangyibin/CPhasing. Accessed 23 July 2025. cited 2025 July 23.

[127] SRA - NCBI. https://www.ncbi.nlm.nih.gov/sra/. Accessed 23 July 2025. cited 2025 July 23.

[128] Keilwagen J, Hartung F, Grau J. GeMoMa: Homology-Based gene prediction utilizing intron position conservation and RNA-seq data. In: Kollmar M, editor. Gene predict methods Protoc [Internet]. New York, NY: Springer; 2019. pp. 161–77. [cited 2025 July 23]. 10.1007/978-1-4939-9173-0_9. 10.1007/978-1-4939-9173-0_9 31020559

[129] Kallenborn F, Chacon A, Hundt C, Sirelkhatim H, Didi K, Dallago C, et al. GPU-accelerated homology search with MMseqs2. BioRxiv; 2024. p. 2024.11.13.623350. 10.1038/s41592-025-02819-8 10.1038/s41592-025-02819-8 PMC12510879 40968302

[130] Gabriel L, Brůna T, Hoff KJ, Ebel M, Lomsadze A, Borodovsky M, et al. BRAKER3: fully automated genome annotation using RNA-seq and protein evidence with GeneMark-ETP, AUGUSTUS, and TSEBRA. Genome Res Cold Spring Harbor Lab. 2024;34:769–77. 10.1101/gr.278090.123. 10.1101/gr.278090.123 PMC11216308 38866550

[131] Brůna T, Lomsadze A, Borodovsky M. GeneMark-ETP significantly improves the accuracy of automatic annotation of large eukaryotic genomes. Genome Res. 2024;34:757–68. 10.1101/gr.278373.123. 38866548 10.1101/gr.278373.123 PMC11216313

[132] Stanke M, Diekhans M, Baertsch R, Haussler D. Using native and syntenically mapped cDNA alignments to improve de Novo gene finding. Bioinforma Oxf Engl. 2008;24:637–44. 10.1093/bioinformatics/btn013. 10.1093/bioinformatics/btn013 18218656

[133] Palmer JM, Stajich J. Funannotate v1.8.1: eukaryotic genome annotation. Zenodo. 2020. 10.5281/zenodo.4054262.

[134] Bolger AM, Lohse M, Usadel B. Trimmomatic: a flexible trimmer for illumina sequence data. Bioinformatics. 2014;30:2114–20. 10.1093/bioinformatics/btu170. 24695404 10.1093/bioinformatics/btu170 PMC4103590

[135] Grabherr MG, Haas BJ, Yassour M, Levin JZ, Thompson DA, Amit I, et al. Full-length transcriptome assembly from RNA-Seq data without a reference genome. Nat Biotechnol. 2011;29:644–52. 10.1038/nbt.1883. 21572440 10.1038/nbt.1883 PMC3571712

[136] Haas BJ, Delcher AL, Mount SM, Wortman JR, Smith RK, Hannick LI, et al. Improving the Arabidopsis genome annotation using maximal transcript alignment assemblies. Nucleic Acids Res. 2003;31:5654–66. 10.1093/nar/gkg770. 14500829 10.1093/nar/gkg770 PMC206470

[137] Kim D, Paggi JM, Park C, Bennett C, Salzberg SL. Graph-based genome alignment and genotyping with HISAT2 and HISAT-genotype. Nat Biotechnol Nat Publishing Group. 2019;37:907–15. 10.1038/s41587-019-0201-4. 10.1038/s41587-019-0201-4 PMC7605509 31375807

[138] Bray NL, Pimentel H, Melsted P, Pachter L. Near-optimal probabilistic RNA-seq quantification. Nat Biotechnol Nat Publishing Group. 2016;34:525–7. 10.1038/nbt.3519. 10.1038/nbt.3519 27043002

[139] Majoros WH, Pertea M, Salzberg SL. TigrScan and glimmerhmm: two open source Ab initio eukaryotic gene-finders. Bioinforma Oxf Engl. 2004;20:2878–9. 10.1093/bioinformatics/bth315. 10.1093/bioinformatics/bth315 15145805

[140] Korf I. Gene finding in novel genomes. BMC Bioinformatics. 2004;5:59. 10.1186/1471-2105-5-59. 15144565 10.1186/1471-2105-5-59 PMC421630

[141] Haas BJ, Salzberg SL, Zhu W, Pertea M, Allen JE, Orvis J, et al. Automated eukaryotic gene structure annotation using evidencemodeler and the program to assemble spliced alignments. Genome Biol. 2008;9:R7. 10.1186/gb-2008-9-1-r7. 18190707 10.1186/gb-2008-9-1-r7 PMC2395244

[142] Karbstein K, Choudhary N, Xie T, Tomasello S, Wagner ND, Barke BH, et al. Efficient assembly of plant genomes: A case study with evolutionary implications in ranunculus (Ranunculaceae). bioRxiv; 2024. p. 2023.08.08.552429. 10.1111/tpj.70390

[143] Testa AC, Hane JK, Ellwood SR, Oliver RP. CodingQuarry: highly accurate hidden Markov model gene prediction in fungal genomes using RNA-seq transcripts. BMC Genomics. 2015;16:170. 10.1186/s12864-015-1344-4. 25887563 10.1186/s12864-015-1344-4 PMC4363200

[144] Vuruputoor VS, Monyak D, Fetter KC, Webster C, Bhattarai A, Shrestha B, et al. Welcome to the big leaves: best practices for improving genome annotation in non-model plant genomes. Appl Plant Sci. 2023;11:e11533. 10.1002/aps3.11533. 37601314 10.1002/aps3.11533 PMC10439824

[145] Woldesemayat AA, Ntushelo K, Modise DM. Identification and characterization of protein coding genes in Monsonia (Monsonia Burkeana Planch. Ex harv) using a combination of approaches. Genes Genomics. 2017;39:245–59. 10.1007/s13258-016-0499-y.

[146] Holst F, Bolger AM, Kindel F, Günther C, Maß J, Triesch S, et al. Helixer: Ab initio prediction of primary eukaryotic gene models combining deep learning and a hidden Markov model. Nat Methods Nat Publishing Group. 2025;1–8. 10.1038/s41592-025-02939-1. 10.1038/s41592-025-02939-1 PMC13076211 41286201

[147] Gabriel L, Becker F, Hoff KJ, Stanke M. Tiberius: end-to-end deep learning with an HMM for gene prediction. Bioinformatics. 2024;40:btae685. 10.1093/bioinformatics/btae685. 39558581 10.1093/bioinformatics/btae685 PMC11645249

[148] Robinson JT, Thorvaldsdottir H, Turner D, Mesirov JP. igv.js: an embeddable javascript implementation of the integrative genomics viewer (IGV). Bioinformatics. 2023;39:btac830. 10.1093/bioinformatics/btac830. 36562559 10.1093/bioinformatics/btac830 PMC9825295

[149] Ou S, Su W, Liao Y, Chougule K, Agda JRA, Hellinga AJ, et al. Benchmarking transposable element annotation methods for creation of a streamlined, comprehensive pipeline. Genome Biol. 2019;20:275. 10.1186/s13059-019-1905-y. 31843001 10.1186/s13059-019-1905-y PMC6913007

[150] Nawrocki EP, Eddy SR. Infernal 1.1: 100-fold faster RNA homology searches. Bioinformatics. 2013;29:2933–5. 10.1093/bioinformatics/btt509. 24008419 10.1093/bioinformatics/btt509 PMC3810854

[151] Chan PP, Lin BY, Mak AJ, Lowe TM. tRNAscan-SE 2.0: improved detection and functional classification of transfer RNA genes. Nucleic Acids Res. 2021;49:9077–96. 10.1093/nar/gkab688. 34417604 10.1093/nar/gkab688 PMC8450103

[152] Nawrocki E. Structural RNA homology search and alignment using covariance models. Theses Diss ETDs. 2009;256. 10.7936/K78050MP.

[153] Lagesen K, Hallin P, Rødland EA, Staerfeldt H-H, Rognes T, Ussery DW. RNAmmer: consistent and rapid annotation of ribosomal RNA genes. Nucleic Acids Res. 2007;35:3100–8. 10.1093/nar/gkm160. 17452365 10.1093/nar/gkm160 PMC1888812

[154] Tillich M, Lehwark P, Pellizzer T, Ulbricht-Jones ES, Fischer A, Bock R, et al. GeSeq – versatile and accurate annotation of organelle genomes. Nucleic Acids Res. 2017;45:W6–11. 10.1093/nar/gkx391. 28486635 10.1093/nar/gkx391 PMC5570176

[155] Shi L, Chen H, Jiang M, Wang L, Wu X, Huang L, et al. CPGAVAS2, an integrated plastome sequence annotator and analyzer. Nucleic Acids Res. 2019;47:W65–73. 10.1093/nar/gkz345. 31066451 10.1093/nar/gkz345 PMC6602467

[156] Li J, Ni Y, Lu Q, Chen H, Liu C. PMGA: A plant mitochondrial genome annotator. Plant Commun. 2025;6:101191. 10.1016/j.xplc.2024.101191. 39521957 10.1016/j.xplc.2024.101191 PMC11956084

[157] Pucker B. Functional Annotation – How to tackle the bottleneck in plant genomics. Preprints; 2024. 10.20944/preprints202402.0645.v1.

[158] Altschul SF, Gish W, Miller W, Myers EW, Lipman DJ. Basic local alignment search tool. J Mol Biol. 1990;215:403–10. 10.1016/S0022-2836(05)80360-2. 2231712 10.1016/S0022-2836(05)80360-2

[159] Buchfink B, Xie C, Huson DH. Fast and sensitive protein alignment using DIAMOND. Nat methods. Nat Publishing Group. 2015;12:59–60. 10.1038/nmeth.3176. 10.1038/nmeth.3176 25402007

[160] Pucker B, Holtgräwe D, Sörensen TR, Stracke R, Viehöver P, Weisshaar B. A de Novo genome sequence assembly of the Arabidopsis Thaliana accession Niederzenz-1 displays Presence/Absence variation and strong synteny. PLOS ONE Public Libr Sci. 2016;11:e0164321. 10.1371/journal.pone.0164321. 10.1371/journal.pone.0164321 PMC5053417 27711162

[161] Jones P, Binns D, Chang H-Y, Fraser M, Li W, McAnulla C, et al. InterProScan 5: genome-scale protein function classification. Bioinformatics. 2014;30:1236–40. 10.1093/bioinformatics/btu031. 24451626 10.1093/bioinformatics/btu031 PMC3998142

[162] Rempel A, Choudhary N, Pucker B. KIPEs3: automatic annotation of biosynthesis pathways. PLOS ONE Public Libr Sci. 2023;18:e0294342. 10.1371/journal.pone.0294342. 10.1371/journal.pone.0294342 PMC10653506 37972102

[163] Mistry J, Chuguransky S, Williams L, Qureshi M, Salazar GA, Sonnhammer ELL, et al. Pfam: the protein families database in 2021. Nucleic Acids Res. 2021;49:D412–9. 10.1093/nar/gkaa913. 33125078 10.1093/nar/gkaa913 PMC7779014

[164] Hunter S, Apweiler R, Attwood TK, Bairoch A, Bateman A, Binns D, et al. InterPro: the integrative protein signature database. Nucleic Acids Res. 2009;37:D211–5. 10.1093/nar/gkn785. 18940856 10.1093/nar/gkn785 PMC2686546

[165] Jensen LJ, Julien P, Kuhn M, von Mering C, Muller J, Doerks T, et al. EggNOG: automated construction and annotation of orthologous groups of genes. Nucleic Acids Res. 2008;36:D250–4. 10.1093/nar/gkm796. 17942413 10.1093/nar/gkm796 PMC2238944

[166] The UniProt Consortium. UniProt: the universal protein knowledgebase in 2023. Nucleic Acids Res. 2023;51:D523–31. 10.1093/nar/gkac1052. 36408920 10.1093/nar/gkac1052 PMC9825514

[167] Rawlings ND, Barrett AJ. MEROPS: the peptidase database. Nucleic Acids Res. 2000;28:323–5. 10.1093/nar/28.1.323. 10592261 10.1093/nar/28.1.323 PMC102440

[168] Siva Shanmugam NR, Yin Y. CAZyme3D: A database of 3D structures for Carbohydrate-active enzymes. J Mol Biol. 2025;437:169001. 10.1016/j.jmb.2025.169001. 39961523 10.1016/j.jmb.2025.169001 PMC13091656

[169] Ashburner M, Ball CA, Blake JA, Botstein D, Butler H, Cherry JM, et al. Gene ontology: tool for the unification of biology. Nat Genet Nat Publishing Group. 2000;25:25–9. 10.1038/75556. 10.1038/75556 PMC3037419 10802651

[170] Wilkinson MD, Dumontier M, Aalbersberg IJ, Appleton G, Axton M, Baak A, et al. The FAIR guiding principles for scientific data management and stewardship. Sci Data Nat Publishing Group. 2016;3:160018. 10.1038/sdata.2016.18. 10.1038/sdata.2016.18 PMC4792175 26978244

[171] Hafner A, DeLeo V, Deng CH, Elsik CG, Fleming S, Harrison D. Data reuse in agricultural genomics research: challenges and recommendations. GigaScience. 2025;14:giae106. 10.1093/gigascience/giae106. 39804724 10.1093/gigascience/giae106 PMC11727710

[172] Sielemann K, Hafner A, Pucker B. The reuse of public datasets in the life sciences: potential risks and rewards. PeerJ. 2020;8:e9954. 10.7717/peerj.9954. 33024631 10.7717/peerj.9954 PMC7518187

[173] ENA - European. Nucleotide Archive. https://www.ebi.ac.uk/ena/browser/home. Accessed 23 July 2025. cited 2025 July 23.

[174] Sequence Read Archive. 2025. https://www.ddbj.nig.ac.jp/dra/index-e.html. Accessed 23 July 2025. cited 2025 July 23.

[175] Dainat J, Hereñú D, Murray DKD, Davis E, Ugrin I, Crouch K et al. NBISweden/AGAT: AGAT-v1.4.1. Zenodo; 2024. 10.5281/zenodo.13799920

[176] Geib SM, Hall B, Derego T, Bremer FT, Cannoles K, Sim SB. Genome annotation generator: a simple tool for generating and correcting WGS annotation tables for NCBI submission. GigaScience. 2018;7:giy018. 10.1093/gigascience/giy018. 29635297 10.1093/gigascience/giy018 PMC5887294

[177] Norling M, Jareborg N, Dainat J. EMBLmyGFF3: a converter facilitating genome annotation submission to European nucleotide archive. BMC Res Notes. 2018;11:584. 10.1186/s13104-018-3686-x. 30103816 10.1186/s13104-018-3686-x PMC6090716

[178] Submission Portal | NCBI | NLM | NIH. https://submit.ncbi.nlm.nih.gov/. Accessed 23 July 2025. cited 2025 July 23.

[179] Webin command line submission interface (Webin-CLI), European A. 2025. https://github.com/enasequence/webin-cli. Accessed 23 July 2025. cited 2025 July 23.

