# Supplementary material for: Cookbook for plant genome sequences
Source: BMC Genomics. 2026 Feb 6;27:231. doi: 10.1186/s12864-026-12623-z (PMC12931034; doi:10.1186/s12864-026-12623-z)
Supplement: Supplementary file 1 — Supplementary Material 1. [file 12864_2026_12623_MOESM1_ESM.pdf]

Cookbook for Plant Genome Sequences

Command-Line Protocols for Plant Genome Sequencing Analysis Using Oxford Nanopore Technologies (ONT) data

de Oliveira, Choudhary, Meckoni *et al*.

Overview

This document provides exemplary step-by-step commands for ONT long-read sequencing analysis in plants, including basecalling, genome assembly, gene annotation, and final data submission. This hands-on protocol covers the entire process, from raw data acquisition to final genome assembly, making it accessible even to beginners. Brief explanations accompany each step to ensure that readers cannot only follow the protocol but also understand the methodology and can adapt it to their own research.

Before introducing the bioinformatics protocol (Steps 1–12), we first describe some basic Unix behavior and software installation requirements (Chapters I and II).

I. General Conventions

Before starting, please note the following basic conventions:

- All commands are intended to be run on a Unix-based/-like system via a command line interface (CLI).
- Commands are generally written on a single line; however, for readability, longer commands may be split across multiple lines using a backslash `\` at the end of each line. When entered into the CLI, these commands will execute as a single line.
- `#` denotes a non-executable comment.
- Example file paths are written as `/path/to`, which should be replaced with the full path to your specific file or directory.
- Commands may include output redirection, e.g., `>> /path/to/logfile.log 2>&1` at the end to redirect both standard output (stdout) and standard error (stderr) to a log file, or `2>/path/to.err.txt` to redirect only stderr to a specified file. Some programs, such as Dorado or samtools, output results to stdout, so it is necessary to redirect these outputs to a file. Using `>` will overwrite the file, while `>>` appends to it.
- The symbol `&` at the end of the command runs it in the background. Alternatively, you may use a terminal multiplexer like `tmux` (https://github.com/tmux/tmux/wiki), which may be more convenient for managing long-running processes.
- If two commands are separated by:
  - `|`: stdout of the first command will be used as an input for the second command (if the second command supports it)
  - `&&`: second command is only executed after the first command finishes without errors
  - `;`: second command is executed after the first command finishes, with or without errors

Links to official documentation are provided for every tool, as they often include many more features and options that cannot be covered in this document. While the examples provided here are based on our experiences, we strongly recommend consulting the official documentation and built-in help to explore options best suited for your specific project.

Helpful options to access tool documentation include:

```
man TOOL                    # Opens the manual page, if available
TOOL -h                     # Prints a short help message
TOOL --help                 # Prints a more extensive help message
cd TOOL && nano README.md   # Many tools provide a README file in the installation directory
```

It is advisable to install and use the latest stable version of each tool to ensure optimal performance and compatibility.

II. Recommended Installation of Tools:

There are several common ways to install bioinformatics tools:

- **Package Manager**: Tools can be installed via system package managers like apt (Debian/Ubuntu), though these versions may be outdated.
- **Precompiled binaries**: Download pre-built binaries from official websites or GitHub repositories.
- **Python environments**: Use `conda` to create isolated environments with specific versions of tools and their dependencies.
- **Docker containers**: Containers typically include all necessary dependencies and are highly reproducible, but they tend to consume more disk space.

The preferred installation method depends on tool availability and user preference. The following sections provide basic usage instructions for each method.

Installation with apt

On Debian/Ubuntu systems, `apt` can be used to install some bioinformatics tools. Basic usage includes:

```
sudo apt update                         # Update package lists
sudo apt upgrade                        # Upgrade installed packages
sudo apt search samtools                # Search for available packages
sudo apt install samtools seqkit        # Install one or more tools
```

Depending on your system configuration, you may need superuser rights (using sudo).

Manual Installation

If no package or conda environment is available, you can manually install a tool. Usually, tool developers provide clear installation instructions, typically involving downloading a binary release and extracting it:

Using pre-compiled Binaries:

```
tar -xvzf tool.tar.xz
cd tool/
```

If binaries are not working or the latest development version is needed, you can compile from source:

```
git clone https://github.com/author/tool.git
cd tool/
make # Follow instructions in the README or INSTALL.md
```

Usually, the full path to the executable binary is required to run the tool. To make the tool accessible in your shell environment, you need to add it to your `PATH` variable. For the current bash session:

```
export PATH="/path/to/tool:$PATH"
```

To make this change permanent across all bash sessions, add the line to your shell startup script (e.g., `~/.bashrc`). To do this, you can use a text editor like `nano`:

```
nano ~/.bashrc
# Add the following line at the end of the file
export PATH="/path/to/tool:$PATH"
# Save and exit, then reload
source ~/.bashrc
```

Python environments

As many bioinformatic tools can be installed via `conda`, we use this package manager to demonstrate virtual environments which can exist along and which can have different versions of the same package. Some tools are dependent on a package (dependency) while another tool might be dependent on the same package, but with another, putatively conflicting, version. The virtual environments can handle these differences. Additionally, you do not need to export the path variable to run a command installed via conda in a virtual environment. Conda installation guide: https://docs.conda.io/projects/conda/en/stable/user-guide/install/linux.html

Common commands, listing existent environments, creating and entering environments, installation and updating:

```
conda env list                          # List existing environments
conda create -n YOUR_ENV_NAME           # Create a new environment
conda activate YOUR_ENV_NAME            # Activate the environment
conda deactivate                        # Deactivate the current environment
conda install TOOL                      # Install a tool in the active environment
conda update -n YOUR_ENV_NAME TOOL      # Update a specific tool
conda update -n YOUR_ENV_NAME --all     # Update all tools in the environment
conda update -n base conda              # Update conda itself
```

Docker containers

Official Docker [1] installation guide: https://docs.docker.com/engine/install/

Docker can run tools in isolated containers. An example BUSCO command:

```
sudo docker run --rm \
 -v /path/to:/path/to \
 ezlabgva/busco:v6.0.0_cv1 \
 busco --plot /path/to/busco_runs
```

In this example

- `-v /path/to:/path/to` mounts your local directory inside the container, making files accessible.
- `ezlabgva/busco:v6.0.0_cv1` specifies the Docker image and version.
- Everything after the image name (`busco --plot ...`) works similarly to a local installation of the same tool.

Most software projects provide ready-to-use Docker images. Using Docker can simplify installation but requires more disk space.

Use Cases

This workflow can be used in two main scenarios:

Sequencing and assembling a new genome: In this case, follow Steps 1–12, starting from raw read acquisition (wet lab steps). Please note that Steps 1–4, which include the laboratory protocols for DNA extraction, library preparation, and sequencing, are already covered in detail in the main manuscript. Hence, this manual includes only brief notes for these steps.

Practicing or working with public data: If you only want to try the bioinformatic workflow using public ONT reads (i.e., no own sequencing), begin with Step 0 (Data Download). If using raw POD5 signal data, proceed to Step 5 for basecalling. If using already basecalled FASTQ files, you can skip directly to Step 6 (Read correction).

Step 0: Downloading Read Data

Public ONT datasets can be used to practice the protocol. In this example, we use *Victoria cruziana* read data [2], which is publicly available on NCBI's Sequence Read Archive (SRA) [3] and the European Nucleotide Archive (ENA)

[4].

0.1 Downloading Raw, Un-basecalled Data

Raw ONT data in POD5 format contains the raw electrical signal from the sequencer. This is the starting point for basecalling, but such data is not always easy to find, as it's not indexed or displayed as clearly as FASTQ files. Therefore, authors should take care to point to the possibility of available raw data in their data availability statements. One possibility is the upload of the data as a compressed archive (.tar.gz,) to ENA which can then be accessed via ENA's ftp interface, documented here: https://ena-docs.readthedocs.io/en/latest/retrieval/file-download.html

ENA hosts such files in this FTP structure: `ftp://ftp.sra.ebi.ac.uk/vol1/run/`

To view or browse these folders in a web browser: `https://ftp.sra.ebi.ac.uk/vol1/run/`

Exemplary, files for read ID `ERR14671536` can be accessed through the ftp interface through a subfolder containing the first few letters of the ID: `ftp://ftp.sra.ebi.ac.uk/vol1/run/ERR146/ERR14671536/`

Once you identify the `.tar.gz` archive, copy the path and download and unpack it using:

```
wget https://ftp.sra.ebi.ac.uk/vol1/run/ERR146/ERR14671536/R252_487107af.tar.gz
tar -xvzf R252_487107af.tar.gz
```

The `tar -xvzf` command unpacks the archive. You will get a folder structured like this:

```
R252_487107af/
|-- fastq/
|   `-- R252_487107af.fastq.gz   # Basecalled data, if already included
`-- pod5/
    |-- FBA42430_skip_487107af_556d06fa_0.pod5
    |-- FBA42430_skip_487107af_556d06fa_1.pod5
    `-- ...
```

The `fastq/` folder contains basecalled data while the `pod5/` folder contains raw signal data that can be used for basecalling.

0.2 Downloading Basecalled Reads with Prefetch and Fasterq-dump

It is also possible to directly download the basecalled reads in fastq format using SRA-Toolkit [5].

SRA-Toolkit Wiki: https://github.com/ncbi/sra-tools/wiki

The recommended tools to download the data are `prefetch` and `fasterq-dump` from the SRA Toolkit.

- `prefetch` downloads data in compressed `.sra` format.
- `fasterq-dump` converts `.sra` files to standard FASTQ format.

First, use `prefetch` to download the `.sra` files. You can specify one or multiple SRA run IDs (e.g., all IDs from BioProject PRJEB63973). Common options include:

- `-p` to show progress,
- `--max-size` to set the maximum size for downloads,
- `--output-directory` to define where downloaded files are stored.

```
prefetch -p \
 --max-size 250g \
 --output-directory /path/to/raw_reads/prefetch \
 ERR13955440 ERR13955441 ERR13955442 [...]
```

Next, extract FASTQ files from the downloaded .sra files using fasterq-dump.

- `e` specifies the number of parallel threads (adjust based on your available CPU cores),
- `--outdir` specifies where to write the FASTQ files.

```
fasterq-dump -e 30 /path/to/raw_reads/prefetch/*/*.sra --outdir /path/to/raw_reads
```

Step 1: Preparations for a Plant Genomics Project

Before beginning a sequencing project, it is important to confirm that genome assemblies or raw sequencing reads are not already available for the species of interest. If new sequencing is required, one of the first steps is to estimate the genome size to plan data requirements accordingly.

The Plant DNA C-values (https://cvalues.science.kew.org/) [6] database can be consulted to obtain the 1C value, which represents the amount of DNA in a haploid genome. For example, the 1C value for Victoria cruziana is reported to be 4.10 picograms (pg). Since 1 pg = 978 megabase pairs (Mbp), this translates to an estimated genome size of: 4.10 pg × 978 Mbp/pg = 4.009 Gbp.

To achieve a 30× sequencing coverage, the minimum amount of raw data required would be: 4.009 Gbp × 30 = ~120.27 Gbp.

However, since part of the data is typically lost during read correction and genome assembly, it is recommended to aim for higher coverage, ideally above 30×.

Refer to Step 1 in the main manuscript for more details about the project planning and preparation considerations.

Step 2: DNA Extraction

The success of long-read sequencing is strongly dependent on the extraction of high-quality, high-molecular-weight (HMW) DNA. DNA integrity and purity directly affect read length and sequencing yield.

Refer to Step 2 in the main manuscript for practical guidance on DNA extraction methods suitable for ONT sequencing, including strategies to minimize shearing and contamination.

Step 3: Library Preparation

Library preparation should follow the manufacturer's instructions, as protocols may vary depending on the type of flow cell (e.g., R9.4.1, R10.4.1), the chemistry kit, or whether native or cDNA protocols are used.

Refer to ONT's updated documentation for the latest protocols and best practices for preparing sequencing libraries.

Step 4: Pore-C

If the goal is to obtain a chromosome-scale genome assembly, long-range information is necessary. In such cases, chromatin conformation capture techniques like Pore-C can be used to scaffold contigs into chromosome-level assemblies by providing information about physical proximity of genomic regions in 3D space.

Refer to Step 4 in the main manuscript for more background.

Step 5: Basecalling

Dorado [7] documentation: https://github.com/nanoporetech/dorado

Basecalling refers to converting raw signal data from ONT sequencers into nucleotide sequences. We use Oxford Nanopore's Dorado, which provides GPU-accelerated basecalling with options for detecting modified bases.

Before basecalling, download the appropriate model for your sequencing chemistry using `dorado download`. You can list available models and options with `dorado basecaller --help`. In this example, we use the `dna_r10.4.1_e8.2_400bps_sup@v5.0.0` model, which includes modified base detection for 5-methylcytosine

(5mCG) and 5-hydroxymethylcytosine (5hmCG). The basecalling input is the folder containing your raw `.pod5` files, and the output is a BAM file. Logs are redirected to an error log file.

```
dorado basecaller \
 dna_r10.4.1_e8.2_400bps_sup@v5.0.0 \
 /path/to/R252_487107af/pod5/ \
 --modified-bases 5mCG_5hmCG \
 > /path/to/Victoria_cruziana_run01.mod.bam \
 2> /path/to/YourArchiveForBasecalling.dorado.mod.err.txt &
```

To convert the resulting BAM file into a FASTQ file (commonly used for downstream analyses), use `samtools fastq` [8].

```
samtools fastq -T "*" \
 /path/to/Victoria_cruziana_run01.mod.bam\
 > /path/to/Victoria_cruziana_run01.mod.fastq
```

- T "*" ensures all auxiliary tags in the BAM file are handled properly.

It is advisable to compress FASTQ files to save disk space. `gzip` compression is widely accepted by downstream bioinformatics tools.

```
gzip /path/to/Victoria_cruziana_run01.mod.fastq
```

You can obtain basic quality statistics, including the read N50, using tools like seqkit [9] (https://bioinf.shenwei.me/seqkit/) or a Python script (`FASTQ_stats3.py` ; https://github.com/bpucker/PlantGenomicsGuide/blob/main/FASTQ_stats3.py)

```
seqkit stats -N 50 /path/to/Victoria_cruziana_run01.mod.fastq.gz

python3 /path/to/FASTQ_stats3.py \
--in /path/to/Victoria_cruziana_run01.mod.fastq.gz
```

Step 6: Raw Read Correction with HERRO

HERRO [10] repository: https://github.com/lbcb-sci/herro

Raw long reads from ONT sequencing often include some errors. Correction improves accuracy before assembly. HERRO is integrated into Dorado and applies a two-step correction:

- Step 1 (CPU intensive) computes overlaps between reads,
- Step 2 (GPU intensive) corrects reads based on these overlaps.

While both steps can be conducted in one command, they can also be run separately to be able to utilize different hardware options to accelerate the running time of the tool due to proper hardware. If you have multiple runs for the same sample (e.g., multiple flowcells), you should merge them into a single file before correction. The merged file can remain compressed.

```
cat\
 /path/to/Victoria_cruziana_run01.mod.fastq.gz \
 /path/to/Victoria_cruziana_run02.mod.fastq.gz \
 /path/to/Victoria_cruziana_run03.mod.fastq.gz \
 > /path/to/Victoria_cruziana.fastq.gz &
```

The first step calculates overlaps between reads and outputs a `.paf` file (specified with `--to-paf`) describing the overlaps. The output, by default, is in stdout and needs to be redirected into a file (using `>`). Use `--threads` to optimize performance based on CPU availability. Errors and logs are saved in *.doc.txt.

```
/path/to/dorado correct \
 /path/to/Victoria_cruziana.fastq.gz \
 --to-paf --threads 10 \
 > /path/to/Victoria_cruziana.overlaps.paf \
 2> /path/to/Victoria_cruziana.overlaps.doc.txt &
```

For the second step (GPU intensive), only uncompressed input FASTQ files are supported. To uncompress them simply use `-gunzip /path/to/Victoria_cruziana.fastq.gz`. Add `-k` if you want to keep the original compressed file. Then, run Dorado with HERRO correction. `--form-paf` specifies the overlap file, and corrected reads are output in FASTA format.

```
/path/to/dorado correct /path/to/Victoria_cruziana.fastq \
 --from-paf /path/to/Victoria_cruziana.overlaps.paf \
 > /path/to/Victoria_cruziana.corrected_reads.fasta \
 2> /path/to/Victoria_cruziana.corrected_reads.errors.txt &
```

Step 7: Genome Sequence Assembly

7.1 Shasta

Official Shasta [11] documentation: https://paoloshasta.github.io/shasta/

Shasta is a fast and efficient assembler for ONT long reads. It uses advanced algorithms to assemble genomes quickly while requiring comparatively modest computational resources. Depending on the type of flow cell used (e.g., R9.4.1, R10.4.1) and whether the reads have undergone prior correction, different pre-configured settings (configuration files) should be used to optimize performance. Key parameters are `--input` specifying the input reads (in FASTA or FASTQ format), `--config` for suitable configuration file, `--assemblyDirectory` defines the output folder where the assembly results will be written, and `--threads` sets the number of CPU threads to use. The `.conf` files can be downloaded from here: https://github.com/paoloshasta/shasta/tree/main/conf

```
/path/to/binary/shasta-Linux-0.14.0 \
 --threads 10 \
 --input /path/to/Victoria_cruziana.corrected_reads.fasta \
 --config /path/to/Nanopore-r10.4.1_e8.2-400bps_sup-Herro-Jan2025.conf \
 --assemblyDirectory /path/to/shasta/Vcruz_01
```

It is recommended to add optional arguments `--memoryBacking 2M` and `--memoryMode filesystem` to enable Shasta to back memory allocations with 2 MB huge pages and use the filesystem for temporary storage. This can speed up performance and reduce disk I/O, but may require root or superuser permissions depending on your system setup.

7.2 NextDenovo

Official NextDenovo [12] documentation: https://nextdenovo.readthedocs.io/en/latest/

NextDenovo is a long-read assembler suitable for large genomes. It performs both read correction and assembly. NextDenovo requires a configuration file that specifies runtime parameters, making it easy to reproduce results or adjust settings for different datasets.

To run NextDenovo, you simply provide the configuration file:

```
/path/to/binary/nextDenovo /path/to/nextdenovo/Vcruz_01.cfg
```

The config file can be copied from the documentation. Key parameters in the config file include:

- `input_fofn`: a file listing the paths to input read file
- `input_type`: `raw` for uncorrected read or `corrected` for pre-corrected reads

- `read_type` : specifies the sequencing technology (ONT, PacBio CLR, or PacBio HiFi)
- `genome_size` : estimated genome size, which guides the assembly process, it is advised to adjust parameters regarding computation resources (`-t` and `-p` options).
- `parallel_jobs` : controls how many parallel processes NextDenovo will run
- `workdir` ; directory where intermediate files and the final assembly is stored

Example configuration for *Victoria Cruziana*

```
[General]
job_type = local
job_prefix = nextDenovo
task = all # "assemble" can be used if read correction was already performed
rewrite = yes
deltmp = yes
parallel_jobs = 6
input_type = raw # alternatively "corrected"
read_type = ont # clr, ont, hifi
input_fofn = /path/to/input.fofn # the .fofn is a file containing the input read file paths
workdir = /path/to/nextdenovo/Vcruz_01 # output directory

[correct_option]
read_cutoff = 1k
genome_size = 4g # estimated genome size
sort_options = -m 20g -t 15
minimap2_options_raw = -t 8
pa_correction = 3
correction_options = -p 15

[assemble_option]
minimap2_options_cns = -t 4
nextgraph_options = -a 1
```

Example `.fofn` file listing input FASTQ files:

```
/path/to/Victoria_cruziana.run1.fastq
/path/to/Victoria_cruziana.run2.fastq
/path/to/Victoria_cruziana.run3.fastq
```

7.3 Verkko

Official Verkko [13] documentation: https://github.com/marbl/verkko

Verkko is a versatile assembler that combines multiple data types, including HERRO-corrected ONT reads or PacBio HiFi reads via `--hifi` , ultra-long ONT reads via `--nano` , and Pore-C data via `--porec` .

A minimal Verkko run includes `-d` to set the output directory, `--hifi` to specify HERRO corrected ONT reads (or PacBio HiFi reads), and optional `--nano` or `--porec` for ultra-long ONT (Dorado basecalled reads) or Pore-C reads, respectively. If `--porec` is given, the correct telomere motif should be specified with `--telomere-motif` . Nowak *et al*. (2025, https://doi.org/10.1101/2024.06.15.599162) reported that Pore-C scaffolding produced better results when conducted with alternative tools like CPhasing. Additionally, `--local-memory` and `--local-cpus` to specify the upper limit of memory in GB (default 64) and the number of CPUs (standard all), respectively, can be specified.

```
verkko --par-run 20 128 48 -d /path/to/verkko/Vcruz_01 --local-memory 128 --local-cpus 10 \
 --hifi /path/to/Victoria_cruziana.corrected_reads.fasta
```

The `--par-run` option allows you to modify CPU, memory (GB), and runtime (hours) limits for specific pipeline stages. For example, `--par-run 20 128 48` allocates 27 CPUs, 464 GB RAM, and 48 hours for one of the internal pipeline steps. This is especially helpful if you encounter bottlenecks in particular stages of the assembly.

For more fine-grained control, consult the full list of Verkko parameters with `verkko --help`.

7.4 Hifiasm

Official Hifiasm [14] documentation: https://github.com/chhylp123/hifiasm

Hifiasm is a modern, ultra-fast assembler primarily designed for PacBio HiFi and ONT R10 reads. For ONT reads, it is essential to use the `--ont` option. Hifiasm includes a read correction step and only accepts reads in FASTQ format. Key paramenters are `-t` for defining the number of CPU threads to use, `--ont` to specify ONT reads, and `-o` to specify the output file prefix.

```
hifiasm -t 64 --ont -o ONT.asm /path/to/Victoria_cruziana.fastq.gz
```

Hifiasm automatically outputs primary and alternative assemblies as GFA files, which can be converted to FASTA using tools such as `awk` or `gfatools`. For `gfatools`, only the `.gfa` file is required:

```
gfatools gfa2fa /path/to/ONT.asm*.gfa > /path/to/Victoria_cruziana.genome.fa
```

Step 8: Assembly Evaluation

After running multiple assemblers, it is essential to evaluate their performance and select the most accurate contig-level assembly for downstream scaffolding. The final assembly should be comprehensively assessed to ensure completeness, accuracy, and overall quality.

8.1 Basic Assembly Statistics

To calculate basic statistics of the assembly such as N50, total length, number of contigs, GC content, the lightweight Python script contig_stats.py (https://github.com/bpucker/PlantGenomicsGuide/blob/main/contig_stats3.py) can be used. It can optionally filter out short contigs using `--min_contig_len` which specifies the minimum length (in basepairs) of a contig in the output FASTA.

```
python3 contig_stats3.py \
 --input /path/to/Victoria_cruziana.genome.fa \
 --min_contig_len 10000 \
 --out /path/to/output_directory/
```

8.2 BUSCO (Benchmarking Universal Single-Copy Orthologs)

Documentation: https://busco.ezlab.org/busco_userguide.html

BUSCO evaluates genome completeness by identifying near-universal single-copy orthologs from curated datasets (OrthoDB) [15]. The first step is to select appropriate lineage dataset. To list available dayasets:

```
busco --list-datasets
```

The output will list current datasets which can be automatically downloaded by BUSCO. BUSCO authors suggests to use the most specific datastet to get highest-resolution analysis. For example, the full lineage of *Victoria cruziana* is (`cellular organisms/Eukaryota/Viridiplantae/Streptophyta/Streptophytina/Embryophyta/Tracheophyta/Euphyllophyta/Spermatophyta/Magnoliopsida/Nymphaeales/Nymphaeaceae/Victoria`). Based on currently available OrthoDB v12 [16] datasets, `eukaryota_odb12`, `viridiplantae_odb12`, and `embryophyta_odb12` datasets can be used. Thus, `embryophyta_odb12` is the current best-fitting dataset for *V. cruziana*.

```
- archaea_odb12 [195]
    - euryarchaeota_odb12 [282]
        - methanomicrobia_odb12 [589]
            - methanosarcinaceae_odb12 [971]
```

```
            - methanosarcina_odb12 [1620]
...
    - viridiplantae_odb12 [822]
        - embryophyta_odb12 [2026]
            - eudicotyledons_odb12 [2805]
                - brassicales_odb12 [4311]

...
```

For the actual BUSCO run, the lineage dataset can be specified with the `-l` or `--lineage_dataset` option. `--mode` is mandatory and can be `genome`, `proteins` or `transcriptome` in the case of a genome sequence FASTA, polypeptide sequences FASTA, or CDS FASTA, respectively. `-i` is input FASTA file and recommended, but not mandatory, is the specification of an output folder and an output folder name. We recommend the output folder to be the same per project or machine running BUSCO, with only the output folder name being changed per run. Thus, all run-specific output folders are collected in a single BUSCO output folder (The given examples are using specific run IDs). To speed up the run, more resources (number of CPUs) can be specified with `--cpu` (default `1`).

```
# BUSCO run for an assembly:
busco -i /path/to/Victoria_cruziana.genome.fa -m genome \
 --cpu 10 -l embryophyta_odb12 --out_path /vol/data/sam/BUSCO_runs/ -o SNM_BIR_0079

# BUSCO run for proteins derived from a structural annotation:
busco -i /path/to/Victoria_cruziana.pep.fa -m proteins --cpu 10 \
 -l embryophyta_odb12 --out_path /vol/data/sam/BUSCO_runs/ -o SNM_BIR_0134
```

BUSCO reports “Complete”, “Fragmented”, and “Missing” BUSCOs to assess assembly quality.

8.3 LTR Assembly Index (LAI)

Documentation: https://github.com/oushujun/LTR_retriever LAI [17],[18] measures genome assembly continuity based on intact Long Terminal Repeat Retrotransposons (LTR-RTs). This involves the following steps:

Step 1: Index the genome using GenomeTools (gt)

```
/path/to/gt suffixerator \
 -db /path/to/Victoria_cruziana.genome.fa \
 -indexname Victoria_cruziana.genome.fa \
 -tis -suf -lcp -des -ssp -sds -dna
```

Step 2: Identify candidate LTR elements using similarity searches

```
/path/to/gt ltrharvest \
 -index Victoria_cruziana.genome.fa \
 -minlenltr 100 -maxlenltr 7000 \
 -mintsd 4 -maxtsd 6 \
 -motif TGCA -motifmis 1 \
 -similar 85 -vic 10 -seed 20 \
 -seqids yes > Victoria_cruziana.genome.fa.harvest.scn
```

Step 3: Detects additional LTR candidates using structural features

```
/path/to/LTR_FINDER_parallel \
 -seq Victoria_cruziana.genome.fa \
 -threads 10 -harvest_out -size 1000000 -time 300
```

Step 4: Combine Results

```
cat Victoria_cruziana.genome.fa.harvest.scn Victoria_cruziana.genome.fa.finder.combine.scn \
 > Victoria_cruziana.genome.fa.rawLTR.scn
```

Step 5: LTR_retriver calculates LAI and filters high confidence LTR-RTs.

```
/path/to/LTR_retriever -genome Victoria_cruziana.genome.fa \
 -inharvest Victoria_cruziana.genome.fa.rawLTR.scn -threads 10
```

The final LAI score is reported in the `.out.LAI` file. LAI scores between 0 to 10 indicate 'draft quality', between 10-20 'reference quality' and more than 20 indicates 'gold quality'.

8.4 Mapping-based Genome Size Estimation (MGSE)

Documentation: https://github.com/bpucker/MGSE

MGSE [19] estimates genome size based on k-mer coverage of reference gene sets (e.g., BUSCO genes). MGSE requires the assembly FASTA file and the reads file, or the directory containing multiple files, in FASTQ format. The reference genes can be provided as GFF3 file. MGSE authors state that the BUSCO genes appears to be the best choice for reference genes. For this the `full_table.tsv` is used which can be found in the BUSCO output directory.

```
python3 MGSE3.py --fasta /path/to/Victoria_cruziana.genome.fa \
--fastq /path/to/Victoria_cruziana.fastq.gz \
--out /path/to/MGSE_output/ \
--ref /path/to/BUSCO_genome/run_embryophyta_odb12/full_table.tsv \
--threads 10
```

Alternatively, if you have a BAM file with the reads mapped to the assembly, that can be provided instead of both assembly and reads file. This BAM file can be generated using minimap2 and can be provided with the `--bam` argument.

```
python3 MGSE3.py --bam /path/to/Victoria_cruziana.assembly_reads.bam \
--out /path/to/MGSE_output/ \
--ref /path/to/BUSCO_genome/run_embryophyta_odb12/full_table.tsv \
--threads 10
```

8.5 Merqury

Documentation: https://github.com/marbl/merqury

Merqury [20] performs a reference-free quality assessment using k-mer-based approaches to calculate consensus accuracy (QV score) and completeness. It is important to select the right k-mer size for assessing the quality. If unsure about the right k size, run `best_k.sh` script provided with MERQURY installation. It just requires the `genome_size` in bases (e.g., 3500000000 for *Victoria_cruziana*). Next, Build k-mer database with `meryl` followed by running Merqury.

```
# Calculate the right k size
sh /path/to/MERQURY/best_k.sh 3500000000

# Count k-mers from raw reads using meryl
 meryl k=21 count /path/to/Victoria_cruziana_corrected_reads.fastq \
 output_Victoria_cruziana.meryl >> /path/to/meryl.log 2>&1 &

# Run Merqury to assess assembly quality
 merqury.sh output_Victoria_cruziana.meryl \
 /path/to/Victoria_cruziana.genome.fasta >> /path/to/merqury.log 2>&1 &
```

Higher QV (Phred quality score) indicates fewer errors [21]. See table below for interpreting QVs:

Phred Quality Score	Probability of incorrect base call	Base call accuracy
10	1 in 10	90%
20	1 in 100	99%
30	1 in 1000	99.9%
40	1 in 10,000	99.99%
50	1 in 100,000	99.999%
60	1 in 1,000,000	99.9999%

8.6 QUAST

Documentation: https://quast.sourceforge.net/docs/manual.html#sec2

QUAST [22] provides a comprehensive summary of assembly quality, reporting metrics like N50, number of contigs, GC content, and misassemblies (if a reference genome sequence is provided). For plants, `--eukaryote` should be specified (default is prokaryotes). `--large` should be specified when the genome is larger than 100 Mbp and `--circos` switches on the circos plotting.

```
/path/to/QUAST/quast.py -o /path/to/quast_results/ \
--eukaryote \
--large \
--circos \
--threads 10 \
--labels "Victoria cruziana" #Assembly name to be used in reports. Quotes should be used if
#label name include spaces.
```

If a reference genome sequence is available, it can be added with `--reference` to detect misassemblies.

Step 9: CPhasing (Scaffolding with Pore-C Data)

Official CPhasing documentation: https://wangyibin.github.io/CPhasing/latest/

CPhasing is a tool for chromosome-scale scaffolding of genome assemblies using Pore-C data. It enhances contiguity by leveraging long-range contact information. Key parameters include `-f` for draft assembly file (FASTA), `-pcd` for Pore-C reads which can be gzipped, `-t` for number of cores, `-p` for restriction enzyme site used in Pore-C library preparation, and `-n` for chromosome count as `chromosome basic numbers:ploidy` (e.g., `12:1` for 12 chromosomes, haploid). `-hcr` is an optional argument that specifies that only high confidence regions should be retained for scaffolding.

```
cphasing pipeline -f /path/to/Victoria_cruziana.genome.fa \
 -pcd /path/to/Victoria_cruziana.porec.fastq.gz -t 24 -n 12:1 -hcr -p CATG
```

The output will be written to a new folder created in the current working directory. This directory contains both the improved assembly and diagnostic reports on scaffolding quality.

Step 10. Structural Annotation

10.1 Protein-coding Genes

10.1.1 RNA-seq Read Mapping with HISAT2

Documentation: https://daehwankimlab.github.io/hisat2/

For gene prediction pipelines like GeMoMa or BRAKER3, mapped RNA-seq data provides essential "hints" about gene structures, such as exon-intron boundaries. HISAT2 [23] is a splice-aware aligner that efficiently maps RNA-

seq reads to a genome assembly. An alternative of HISAT2 is STAR [24] (https://github.com/alexdobin/STAR) and can be used instead, if preferred.

Before mapping, HISAT2 requires creating an index from the genome assembly FASTA file. Input genome assembly in FASTA format and output prefix (VC for Victoria_cruziana) for the generated index files are required.

```
hisat2-build -p 10 \ # -p specifies the number of cores
/path/to/Victoria_cruziana.genome.fa VC
```

Indexing creates several auxiliary files (`.ht2` files) that are required for mapping.

After indexing, RNA-seq reads (paired-end or single-end) are aligned to the genome sequence. The input reads can be a comma separated list of file paths and may be gzip compressed. For paired end reads, the arguments `-1` and `-2` are used and for single-end reads `-U` is used. The output can be piped (`|`) to samtools [8] to immediately sort the resulting mapping by genomic coordinates, save it in BAM format and generate an index of it. The output BAM file + index will be used as RNA-seq hint input for downstream annotation tools.

```
hisat2 -p 10 \
 -x /path/to/Victoria_cruziana.genome.fa.index \
 -1 /path/to/RNA-seq_reads_001_1.fq.gz,/path/to/RNA-seq_reads_002_1.fq.gz \
 -2 /path/to/RNA-seq_reads_001_2.fq.gz,/path/to/RNA-seq_reads_002_2.fq.gz \
 | samtools sort - -O BAM | tee /path/to/Victoria_cruziana.genome_RNA-seq_mapping.bam \
 | samtools index - /path/to/Victoria_cruziana.genome_RNA-seq_mapping.bam.bai
```

10.1.2 BRAKER3

Documentation: https://github.com/Gaius-Augustus/BRAKER

BRAKER3 [25] is a fully automated pipeline for annotating protein-coding genes using RNA-seq data and/or protein sequences as hints. It combines multiple gene prediction tools like GeneMark [26] and AUGUSTUS [27]

10.1.2.1 Obtaining Protein Hints Protein hints guide BRAKER3 to make more accurate predictions. The source for these protein hints can be the precompiled protein hints from OrthoDB 12 [16] (https://bioinf.uni-greifswald.de/bioinf/partitioned_odb12/ ; `Viridiplantae`). Additional peptide sequences from UniProt database [28] can be included. Using advanced UniProt search, a broader taxon can be given via the 'Taxonomy' search field. In the case of *Victoria cruziana*, its order is Nymphaeales [261007] (which translates to the UniProt search key "(taxonomy_id:261007)"). Multiple FASTA files as a comma-separated list for the `--prot_seq` parameter can be given.

10.1.2.2 BRAKER3 Command Input arguments are `--genome` for genome assembly in FASTA format, `--threads` to define number of CPU threads to use, `--workingdir` to define the output directory, `--gff3` output annotations in GFF3 format, `--bam` defines path to RNA-seq BAM alignment file, `--prot_seq` protein sequences for hints, and `--busco_lineage` defines the BUSCO lineage dataset for training AUGUSTUS.

```
 /path/to/braker.pl --genome=/path/to/Victoria_cruziana.genome.fa --threads=10 \
 --workingdir=/path/to/braker3/Vcruz_annotation01/ --gff3 \
 --bam=/path/to/Victoria_cruziana.genome_RNA-seq_mapping.bam \
 --prot_seq=/path/to/Viridiplantae.fa,/path/to/Uniprot_hints.fasta \
 --busco_lineage=embryophyta_odb12
```

10.1.3 GeMoMa

Documentation: https://www.jstacs.de/index.php/GeMoMa and https://www.jstacs.de/index.php/GeMoMa-Docs

GeMoMa [29] uses homology-based gene prediction by aligning transcript sequences from closely related reference species and optionally includes RNA-seq evidence. The first step is to obtain reference genome sequences and their structural annotation in GFF/GFF3 file format from closely-related species. For our example *Victoria*

cruziana, closely related species, *Nymphaea colorata* (GenBank ID: GCA_008831285.2) and *Nymphaea thermarum* (GenBank ID: GCA_011799765.1) are used.

In the below example run, each reference species is given with 4 parameters. `s=own` specifies that a custom reference species is given, `i=` is an optional identifier tag for reference and the genome assembly and GFF3 file are provided with `g=` and `a=`, respectively. `t=` is used to specify the path of the target genome sequence, `outdir=` to give the output directory path, and `threads=` for specifying the number of threads to use. `r=MAPPED` is specifying that the RNA-seq hints are mapped and `ERE.m=` specifies the path to the mapping file. `ERE.m=` can be speciefied multiple times. Both of these arguments can be skipped if RNA-seq data is not available. The default output is GFF file only. `pc=true` outputs FASTA coding sequences, `p=true` output FASTA protein sequences `pgr=true` specifies that FASTA genomic regions should be returned, and `o=true` specifies that the predictions per individual reference should also be returned. This is important for further filtering steps. `Extractor.r` specifics that GeMoMa will try to repair transcript annotations that cannot be parsed. `GAF.f` specifies a filter string that filters the output. In the below example, only predictions with proper start (start== `M`) and stop (stop== `*`) codons are kept. Additionally, if a prediction has a certain score to length of amino acid ratio, it will be kept (score/aa>='0.75'). `AnnotationFinalizer.r=NO` option skips the renaming step.

```
java -jar /path/to/GeMoMa-1.9.jar CLI GeMoMaPipeline \
 t=/path/to/Victoria_cruziana.genome.fa \
 s=own i=NyCol a=/path/to/ref_species/GCF_008831285.2/genomic.gff \
 g=/path/to/ref_species/GCF_008831285.2/GCF_008831285.2_ASM883128v2_genomic.fna \
 s=own i=NyTher a=/path/to/ref_species/GCA_011799765.1/genomic.gff \
 g=/path/to/ref_species/GCA_011799765.1/GCA_011799765.1_ASM1179976v1_genomic.fna r=MAPPED \
 ERE.m=/path/to/Victoria_cruziana.genome_RNA-seq_mapping.bam \
 outdir=/path/to/gemoma/Vcruz_annotation02/ \
 pc=true pgr=true p=true o=true Extractor.r=true \
 GAF.f="start=='M' and stop=='*' and (score/aa>='0.75')" \
 AnnotationFinalizer.r=NO threads=10 >> GeMoMa.log 2>&1 \
```

The resulting output directory will have multiple files. The most important files are individual predictions for each reference as a separate prediction GFF file and a combined prediction from all references, generated with the specified filter. In case you do not want to merge external annotations and GeMoMa annotation is already good enough, then you can further filter it (if number of genes are too high) and proceed with the finalizing steps described in section 10.1.5.

10.1.4 Funannotate

Documentation: https://funannotate.readthedocs.io/en/latest/index.html

The Funannotate pipeline [30] can perform gene prediction, functional annotation, and comparison. Here, we will only focus on gene prediction. There are multiple steps to generate a Funannotate prediction.

1. First, optional step is to clean your assembly. If you have a haploid assembly, `funannotate clean` can remove some repetitive small, low-quality contigs:

```
funannotate clean -i /path/to/Victoria_genome.fa \
 -o /path/to/Victoria_cruziana_cleaned.genome.fa \
 -m 500 #min. length of contig to keep, by default 500
```

2. Second mandatory step is to softmask the repetitive elements in the assembly using `tantan`:

```
funannotate mask -i /path/to/Victoria_cruziana_cleaned.genome.fa \
 -o /path/to/Victoria_cruziana_cleaned_softmasked.genome.fa \
 --cpus 10 # No. of cpus to use
```

3. Now, `funannotate train` uses RNA-seq data from the target species and generates genome-guided `Trinity` assembly followed by `PASA` assembly. If RNA-seq data is not available, this step does *de novo* `Augustus` training. It produces the input data for funannotate predict, i.e. coordinate-sorted BAM alignments, trinity transcripts, and high quality PASA GFF3 annotation. Single-end FASTQ files could also be

provided with `-s` argument.

```
funannotate train -i /path/to/Victoria_cruziana_cleaned_softmasked.genome.fa \
 -o /path/to/funannotate_output/ #Output folder \
 -l /path/to/RNA-seq_reads_001_1.fq.gz /path/to/RNA-seq_reads_002_1.fq.gz \
 -r /path/to/RNA-seq_reads_001_2.fq.gz /path/to/RNA-seq_reads_002_2.fq.gz \
 --cpus 10 \
 --species "Victoria cruziana" \
 --max_intronlen 1000000
```

4. The next step is to use `funannotate predict` to predict gene models. It automatically uses the results from the `train` step and incorporates them into the prediction, given the same output directory as `train` step. Additional, protein evidence from closely related species can be used with `--protein_evidence` argument.

```
funannotate predict -i /path/to/Victoria_cruziana_cleaned_softmasked.genome.fa \
 -o /path/to/funannotate_output/ #Output folder \
 -s "Victoria cruziana" --busco_db embryophyta \
 --organism other \
 --protein_evidence /path/to/ref_species/GCA_008831285.2/peptide.fasta \
 /path/to/ref_species/GCA_011799765.1/peptide.fasta \
 --cpus 10 \
 --max_intronlen 1000000
```

5. Finally, `funannotate update` command can be used to add UTRs and refine gene models RNA-seq data.

```
funannotate update -i /path/to/funannotate_predict/ \
 --species "Victoria_cruziana" \
 -o path/to/funannotate_output/ \
 --cpus 10
```

10.1.5 Helixer and Tiberius

Documentation: https://github.com/usadellab/Helixer

Documentation: https://github.com/Gaius-Augustus/Tiberius

Helixer [31] and Tiberius [32] are *ab initio* gene prediction tools that are utilising deep learning.

10.1.5.1 Helixer

1. Get the latest models:

```
Helixer/scripts/fetch_helixer_models.py -c /vol/data/tools/helixer-models
```

2. Run Helixer:

```
Helixer.py \
 --model-filepath /vol/data/tools/helixer-models/land_plant/land_plant_v0.3_a_0080.h5 \
 --fasta-path /path/to/Victoria_genome.fa --species Victoria_cruziana \
 --gff-output-path /path/to/Vcruz_helixer_annotation.gff3 --subsequence-length 64152
```

10.1.5.2 Tiberius

1. Repeat softmasking of the genome sequence assembly with RepeatModeler (e.g. from here: https://github.com/Dfam-consortium/TETools):

```
BuildDatabase -name Vcruziana_db.db /path/to/Victoria_genome.fa
RepeatModeler -database Vcruziana_db.db -threads 27 -LTRStruct
RepeatMasker -pa 24 -lib Vcruziana_db.db-families.fa -xsmall /path/to/Victoria_genome.fa
```

2. Run Tiberius on the softmasked genome sequence assembly:

```
python /path/to/Tiberius2/Tiberius/tiberius.py \
 --model_cfg /path/to/Tiberius2/Tiberius/model_cfg/eudicotyledons.yaml \
 --genome /path/to/Victoria_genome.fa.masked --out /path/to/Vcruz_tiberius_annotation.gtf \
 --no_softmasking --seq_len 259992
```

10.1.6 Combining and Finalizing Annotations Using GeMoMa

It is important to evaluate the quality of annotations from different sources which can vary in quality and completeness. To evaluate the completeness, BUSCO score using same lineage dataset and in protein mode should be calculated. The goal is to get the closest BUSCO score to the genome (same or higher). If any annotation recovers the genome BUSCO, it might be sufficient and do not require merging. However, if that is not the case, evaluating, filtering, and combining these annotations would be beneficial to create a final high-confidence gene set. Below is a step-by-step guide to combine annotations from different sources:

Step 1: Prepare RNA-seq evidence with GeMoMa's ERE

Although all three gene annotation tools described above can incorporate `GFF3` from external sources, GeMoMa has been observed to give the best results. To compare annotations from different sources, they should be compared on the same scale. GeMoMa's gene models have some attributes that define the quality of the gene models. To add these attributes to other annotations, we need to extract RNA-seq coverage and intron information from the RNA-seq alignments. GeMoMa's `ERE` module is used for this where `m=` specifies the input RNA-seq alignment in BAM format sorted by coordinates and `outdir=` specifies the directory where ERE will store its output.

```
java -jar /path/to/GeMoMa-1.9.jar CLI ERE \
 m=/path/to/Victoria_cruziana.genome_RNA-seq_mapping.bam \
 outdir=/path/to/gemoma/Vcruz_RNA_evidence
```

This command creates `coverage.bedgraph` (read coverage across the genome) and `introns.gff` (detected intron positions). These files are used to add quality attributes to gene models based on expression data.

Step 2: Add attributes to external annotations with AnnotationEvidence

Now, gene annotations (e.g., from BRAKER3 or Funannotate) are enriched with RNA-seq based metrics. In the below command, `a=` is the external annotation GFF3 file, `g=` is the genome assembly FASTA file, `c=` defines the coverage file (BAM format and can be `UNSTRANDED`, `STRANDED`, and `NO`). In `UNSTRANDED`, only one `.bedgraph` file is needed, while for `STRANDED`, two `.bedgraph` file are required under `coverage_forward` and `coverage_reverse` attribute. `i=` points to intron evidence, and `outdir=` specifies the output directory. Example with BRAKER3 output:

```
java -jar /path/to/GeMoMa-1.9.jar CLI AnnotationEvidence \
 a=/path/to/braker3/Vcruz_annotation01/braker.gff3 \
 g=/path/to/Victoria_cruziana.genome.fa \
 c=UNSTRANDED \
 coverage_unstranded=/path/to/gemoma/Vcruz_RNA_evidence/coverage.bedgraph \
 i=/path/to/gemoma/Vcruz_RNA_evidence/introns.gff \
 outdir=/path/to/gemoma/Vcruz_braker_anno_with_evidence/
```

This command will produce among other files, the `annotation_with_attributes.gff` file, with the added attributes. Each gene has useful attributes, such as average RNA-seq coverage per gene (`avgCov`), fraction of transcript's introns supported by RNA-seq (`tie`), amino acid sequence length (`aa`) among multiple others. Note that `score` is confidence score for GeMoMa predictions and therefore, external annotations do not have this. These attributes allows objective filtering in the next step based on biological evidence.

Step 3: Filter and merge annotations using GeMoMa GAF

Gene annotations often include low-confidence or biologically implausible predictions (e.g., unsupported isoforms, incomplete genes). GeMoMa's GAF (GeMoMa Annotation Filter) can filter low-quality genes based on RNA-seq support and other metrics, combine multiple annotations (e.g., GeMoMa + BRAKER3 + Funannotate), and remove

redundancy and select the best gene models. In the GeMoMa Annotation Filter (`GAF`) command. `g=` specifies input GFF3 files (can be used multiple times for multiple annotations), `f=` sets the filtering criteria, for example, `isNaN(score)` meaning score is not available, is necessary to include external annotations since they do not have the score attribute. Multiple filtering criteria can be combined with the logical use of `and`, `or` and brackets `()`. `atf=` filters alternative isoforms (optional but recommended). `outdir=` sets the output directory.

```
java -jar /path/to/GeMoMa-1.9.jar CLI GAF\
 g=/path/to/gemoma/Vcruz_braker_anno_with_evidence/filtered_predictions.gff\
 g=/path/to/gemoma/Vcruz_annotation02/unfiltered_predictions_from_species_0.gff\
 g=/path/to/gemoma/Vcruz_annotation02/unfiltered_predictions_from_species_1.gff\
 f="start=='M' and stop=='*' and aa>=15 and avgCov>0 and (isNaN(score) or score/aa>='3.25')"\
 atf="tie==1 or sumWeight>1" outdir=/path/to/gemoma/Vcruz_filter_01
```

In the above command, `start=='M' and stop=='*'` only keeps genes with proper start and stop codons, `aa>=15` removes predictions encoding very short peptides (likely spurious), `avgCov>0` keeps only genes with any RNA-seq support, `is(NaN(score) or score/aa>='3.25')` keeps genes with good normalized scores for GeMoMa genes; for external tools (which don't have a GeMoMa score), the filter allows inclusion by skipping score check. `atf="tie=1 or sumWeight>6"` filters alternate transcripts to retain those with complete intron support (`tie==1`) or strong overall support (`sumWeight>1`). These filters can (and should) be customized based on particular projects. Always check BUSCO scores (protein mode) after filtering steps to validate completeness and investigate annotation statistics (total number of genes and transcripts). It is recommended to iteratively test stricter or more relaxed filter criteria for optimal balance between gene count and completeness.

Step 4: Rename genes using AnnotationFinalizer

Once an annotation is merged and sufficiently filtered (reasonable number of genes and high BUSCO score), it will have inconsistent gene IDs (from different tools and species references). To have standardized gene IDs, GeMoMa's `AnnotationFinalizer` is used. `g=` is genome FASTA, `a=` is filtered annotation GFF3 file, `p=` sets the prefix for all gene IDs, `n=false` disables adding gene IDs as extra name attributes, and `outdir=` defines the output directory.

```
java -jar /path/to/GeMoMa-1.9.jar CLI AnnotationFinalizer \
 g=/path/to/Victoria_cruziana.genome.fa \
 a=/path/to/gemoma/Vcruz_filter_01/filtered_predictions.gff \
 tf=true rename=SIMPLE p=Vcruz_ \
 outdir=/path/to/gemoma/Vcruz_final_01/ n=false
```

After this step, all genes will be named uniformly, e.g., Vcruz_00001, Vcruz_00002, etc.

Step 5: Extract CDS and protein sequences using Extractor

Final annotations are in GFF3 format, to generate protein-coding sequences and peptide FASTA sequences, GeMoMa's `Extractor` can be used. `g=` specifies the genome FASTA file, `a=` specifies the finalized gff3 file, `p=true` and `c=true` outputs protein FASTA and CDS FASTA files, respectively. Output will be written to `outdir=`.

```
java -jar /path/to/GeMoMa-1.9.jar CLI Extractor \
 g=/path/to/Victoria_cruziana.genome.fa a=/path/to/gemoma/Vcruz_final_01/final_annotation.gff \
 p=true c=true
```

10.2 Non-coding Genes

10.2.1 Identification of TE's with EDTA

Documentation:https://github.com/oushujun/EDTA

EDTA (Extensive de-novo TE Annotator) [33] is a pipeline designed to comprehensively annotate transposable elements (TEs) in a genome. `--genome` defines the path to the genome assembly in FASTA format, `--cds` (optional) path to coding sequence FASTA file, this helps EDTA to make gene regions to reduce false-positive TE annotations. `--overwrite 1` allows EDTA to overwrite existing files in the directory (use with caution), `--anno 1` runs

the complete annotation workflow after TE library construction, `--sensitive 1` activates sensitive mode for better detection of complex or nested TEs (slower), `--evaluates 1` performs an evaluation of annotation quality, `--threads` allocated CPU threads for parallel processing.

```
/path/to/EDTA/EDTA.pl --genome /path/to/Victoria_cruziana.genome.fa \
--cds /path/to/Victoria_cruziana.cds.fa \
--overwrite 1 \
--anno 1 \
--sensitive 1 \
--evaluate 1 \
--threads 10
```

10.2.2 Identification of ncRNAs with Infernal

Documentation: http://eddylab.org/infernal

Infernal (INFERence of RNA ALignment) [34] identifies non-coding RNAs using covariance models (CMs), which are statistical models representing RNA secondary structures. Rfam database [35] has pre-available calibrated covariance models required by Infernal's cmscan which can be downloaded from ftp://ftp.ebi.ac.uk/pub/databases/Rfam/CURRENT/Rfam.cm.gz and an additional Claninfo file https://ftp.ebi.ac.uk/pub/databases/Rfam/CURRENT/Rfam.clanin.

```
#Install latest covariance models
wget ftp://ftp.ebi.ac.uk/pub/databases/Rfam/CURRENT/Rfam.cm.gz

#Uncompress Rfam Covariance models
gunzip Rfm.cm.gz

#Index (compress) the Rfam.cm file for faster searching
/path/to/infernal/src/cmpress /path/to/Rfam.cm.gz

#Run cmscan to search the genome for ncRNA matches
/path/to/infernal/src/cmscan --nohmmonly \
--rfam --cut_ga --fmt 2 --oclan --oskip \
--clanin Rfam.clanin -o /path/to/my_cmscan_out \
--tblout /path/to/my_cmscan_tblout \
/path/to/Rfam.cm /path/to/Victoria_cruziana.genome.fa
```

`--nohmmonly` uses covariance models instead of faster but less accurate HMM-only searches, `--rfam` tailors search to Rfam-specific conventions, automatically applying recommended cutoffs, `--cut_ga` filters hits based on gathering thresholds (GA) from Rfam to reduce false positives, `--fmt 2` specifies human-readable format, `--oclan` outputs RNA family clan information, `--oskip` sking saving the full alignment for faster execution, `--clanin` supplies clan information file, `-o` is the output file for full cmscan report, `--tblout` provides tabular summary output file, `/path/to/Rfam.cm` defines input covariance models, and `/path/to/genome.fa` defines input genome to search for ncRNAs

Rfam database is a comprehensive database including sequence datasets of most non-coding RNA families. Hence, different types of ncRNAs can be identified with Infernal search using Rfam covariance model. However, it is also possible to use specialized tools for particular ncRNA's. Few of them are:

- tRNAs:
  (a) tRNAscan-SE [36] : https://github.com/UCSC-LoweLab/tRNAscan-SE
- rRNAs:
  (a) RNAmmer [37] : https://services.healthtech.dtu.dk/services/RNAmmer-1.2/
  (b) SSU-ALIGN [38] : http://eddylab.org/software/ssu-align/

Step 11: Functional Annotation

Functional annotation involves predicting the biological role of each protein-coding gene. This includes assigning functional terms (GO, KEGG), identifying homologs, and classifying proteins into families or pathways.

11.1 Orthlogy-based Annotation via Reciprocal Best Hits (RBH)

Reciprocal Best Hit (RBH) searches provide a reliable method to predict orthologous relationships by identifying gene pairs that are each other's best match. To perform RBH search and transfer functional annotation, it is necessary to have a well-annotated reference. For this, for e.g., Arabidopsis thaliana can be used. The Python script `match_proteins.py` at https://github.com/bpucker/PlantGenomicsGuide/blob/main/match_proteins.py can be used where `--prefix` defines the output directory where results will be saved, `--input1` is the peptide file from target species, `--input2` is the peptide file from reference species, and `--cpu` defines the number of cores for parallel BLAST search (default:8). The `match_proteins.py` script supplements RBHs with best match if no strict RBHs are found.

```
python3 match_proteins.py \
--prefix /path/to/output_directory/ \
--input1 /path/to/Victoria_cruziana.pep.fasta \
--input2 /path/to/Arabidopsis_thaliana.pep.fasta \
--cpus 10
```

An alternative to the above script is the `construct_anno.py` (https://github.com/bpucker/PlantGenomicsGuide/blob/main/construct_anno.py). It provides a more comprehensive one-step workflow that combines the function of above script (sequence similarity search) and annotation transfer.

```
python3 construct_anno.py \
--in /path/to/Victoria_cruziana_pep.fasta \
--out path/to/output_directory \
--ref /path/to/Arabidopsis_thaliana_araport11.pep.fasta \
--anno /path/to/Arabidopsis_thaliana_araport11.anno.txt
```

In the above example command, `--in` specifies the peptide file of target species, `--out` specifies the directory where functional annotation results will be stored, `--ref` specifies the peptide file of reference species, and `--anno` specifies the tab-separated annotation file matching `--ref` file. In the given example *Arabidopsis thaliana* is used [39], but can by replaced by any other species, given the protein file and the corresponding annotation file exists.

11.2 InterProScan

Documentation: https://interproscan-docs.readthedocs.io/en/latest/

InterProScan [40] integrates multiple protein signature databases (Pfam, PRINTS, PROSITE, SMART, etc.) to classify proteins, predict domains, and important sites. To run InterProScan, first obtain a copy of the tool, unpack it, setup and then finally run InterProScan.

```
mkdir interproscan #at your desired location
cd interproscan
wget https://ftp.ebi.ac.uk/pub/software/unix/iprscan/5/5.75-106.0/\
interproscan-5.75-106.0-64-bit.tar.gz
wget https://ftp.ebi.ac.uk/pub/software/unix/iprscan/5/5.75-106.0/\
interproscan-5.75-106.0-64-bit.tar.gz.md5

# Recommended checksum to confirm the download was successful:
md5sum -c interproscan-5.75-106.0-64-bit.tar.gz.md5
# Must return *interproscan-5.75-106.0-64-bit.tar.gz: OK*
# If not - try downloading the file again as it may be a corrupted copy.
```

```
tar -pxvzf interproscan-5.75-106.0-*-bit.tar.gz
cd interproscan-5.75*

#to prepare hmm models into a format used by hmmscan
python3 setup.py -f interproscan.properties

#Run InterProScan
./interproscan.sh -i /path/to/Victoria_cruziana.pep.fasta \
-f xml  -cpu 10 \
-o /path/to/iprscan_results.xml
```

`-i` defines input protein FASTA file, `-o` defines the output file, `-cpu` allocates CPUs, and `-f` defines the output format and can be `xml`, `tsv`, `gff3`, and `json`. If the results are needed for `funannotate annotate` command, `xml` format is necessary.

11.3 Specialized Functional Annotation Tools

There are some functional annotation tools for specific pathway or enzyme-family gene annotation such as:

- KIPEs3 [41] (Flavonoid/Carotenoid biosynthesis): https://github.com/bpucker/KIPEs
- MYB annotator [42] (MYB transcription factors): https://github.com/bpucker/MYB_annotator
- bHLH annotator [43] (bHLH transcription factors): https://github.com/bpucker/bHLH_annotator

11.4 Funannotate Annotate

Documentation: https://funannotate.readthedocs.io/en/latest/index.html

Funannotate [30] integrates multiple annotation sources, formats output for NCBI submission, and assigns functional terms. `--gff3` specifies final structural annotation (gff3 format), `--fasta` specifies genome assembly in FASTA format, `-s` specifies species name, `-o` for output directory, and `--sbt` is optional and used to define the path to the NCBI submission template. This is necessary if NCBI submission is desired, the file can be downloaded from NCBI website after filling basic details about the genome sequencing project (https://submit.ncbi.nlm.nih.gov/genbank/template/submission/). `-a` is an optional custom annotation TSV (gene ID, product name), `--iprscan` is the InterProScan XML file for protein domain annotation, and `--rename` is the LOCUS_TAG that can be specified when starting a WGS submission or is assigned automatically later.

```
funannotate annotate \
--gff /path/to/Victoria_cruziana.gff3 \
--fasta /path/to/Victoria_cruziana.genome.fasta \
-s "Victoria cruziana" \
-o /path/to/funannotate_annotate_output/ \
--sbt /path/to/NCBI_template/ \
-a /path/to/custom_annotations \
--iprscan /path/to/iprscan_results.xml
--rename LOCUS_TAG \
--busco_db embryophyta --cpus 10
```

Step 12: Data Submission

After generating genome assemblies and annotations, it's crucial to share your data through public repositories (e.g., NCBI, ENA, DDBJ) to promote reproducibility and open science. These databases are interconnected through the INSDC and hence, submission to any of the three makes it accessible on all three INSDC repositories, i.e. the NCBI, EMBL, and DDBJ.

Before starting the submission process, it is necessary to register the study (project) and sample. For example, for ENA, this is possible via ENA Webin portal by selecting "Register Study" button and filling out necessary details.

More detailed information can be found here: https://ena-docs.readthedocs.io/en/latest/submit/study/interactive.html.

Once study registration is complete, a study(project) accession number is assigned.

To register samples, similar to study, this can be done through the Webin portal selecting "Register Samples" button. Here, you have to define metadata about the sequenced source material using a metadata spreadsheet. The template of the spreadsheet is available for download in the Webin portal after selecting the most appropriate checklist group. For more details: https://ena-docs.readthedocs.io/en/latest/submit/samples/interactive.html

Once the samples are validated and if accepted, sample accession numbers are assigned.

12.1 Signal Data (POD5) and Read (FASTQ) Submission

Before submitting the reads to ENA, it is necessary that they follow the ENA guidelines. Post basecalling, the fastq headers often have long metadata lines, the script `clean_fastq_headers.py` (https://github.com/bpucker/PlantGenomicsGuide/blob/main/clean_fastq_headers.py) aids in cleaning the headers to make the files ENA-submission ready. `--in` defines the input fastq file (gzip compressed) while the `--out` defines the output fastq file:

```
python3 clean_fastq_headers.py \
 --in Victoria_cruziana_run01.mod.fastq.gz \
 --out VC_run01_cleaned.fastq

gzip VC_run01_cleaned.fastq
```

File submission can be done through ENA FTP server which requires that the files are compressed and their MD5 checksum is registered in lower case letters. ONT native data (POD5 files) and basecalled reads (FASTQ files) should be submitted as a single tar.gz archive. An example directory structure for a run named `VC_01` would be:

```
VC_01/
|-- fastq/
|   `-- VC_run01_cleaned.fastq.gz
`-- pod5/
    |-- VC_run01_1.pod5
    |-- VC_run01_2.pod5
    |-- VC_run01_3.pod5
    `-- ...
```

To archive the entire directory, `tar` command is used, followed by calculating the MD5 value:

```
tar -cvzf VC_01.tar.gz /path/to/VC_01/
md5sum VC_01.tar.gz > VC_01.tar.gz.md5
```

Finally, the file can be submitted via FTP command providing the webin login credentials:

```
ftp webin.ebi.ac.uk
WEBIN-XXXXX
XXXXXXXXX
mput VC_01.tar.gz
mput VC_01.tar.gz.md5
bye
```

where `ftp` establishes a connection to the ENA through the terminal, followed by entering `Webin-username` and `Password` when prompted. `mput <filename>` uploads the file and `bye` command exits the ftp client.

12.2 Genome Assembly and Annotation Submission

Depending on whether you have only a genome assembly or also annotation (gene predictions), the submission formats and requirements vary slightly for ENA and NCBI (INSDC databases).

Before submission, it is crucial to validate and clean the annotation files (typically in GFF3 format), as submission portals like ENA and NCBI have strict validation criteria. Common issues include duplicated feature locations and formatting inconsistencies. We recommend using the AGAT toolkit [44] for this purpose:

```
agat_convert_sp_gxf2gxf.pl \
 -g /path/to/Victoria_cruziana.gff3 \
 -o /path/to/Victoria_cruziana_standard.gff3
```

`-g` defines input GFF3 annotation file and `o` is the output standardized GFF3 file

Then, fix any duplicated feature locations:

```
agat_sp_fix_features_locations_duplicated.pl \
 -f /path/to/Victoria_cruziana_standard.gff3 \
 -o /path/to/Victoria_cruziana_standard_deduplicated.gff3
```

`-f` is the input standardized GFF3 file and `-o` is the output GFF3 file with duplicated feature locations removed.

12.2.1 Submission to EMBL-EBI's ENA

Documentation: https://ena-docs.readthedocs.io/en/latest/submit/assembly/genome.html

Step 1: Convert to flat file format

For submitting genome assembly along with annotations to ENA, you must prepare a "flat file" format (*.embl file), which combines the genome sequence and annotation in a single file. We recommend using the tool `EMBLmyGFF3` [45] (https://github.com/NBISweden/EMBLmyGFF3). It takes as positional arguments the `gff` file and the `FASTA` file as well as some metadata and writes the output to a specified file (`-o`).

```
EMBLmyGFF3 /path/to/Victoria_cruziana_standard.gff3 /path/to/Victoria_cruziana.genome.fa \
 --topology linear --molecule_type 'genomic DNA' --transl_table 1 \
 --species 'Victoria cruziana' --locus_tag LOCUSTAG \
 --project_id PRJXXXXXXX -o /path/to/Vcruz.embl
```

Step 2: Compress the flat file

ENA requires gzipped flat files for submission. For efficient compression using multiple CPU threads, `pigz` (https://github.com/madler/pigz) tool can be used. The -k option is optional and keeps the original file.

```
pigz -k /path/to/Vcruz.embl
```

Step 3: Prepare the manifest file

The manifest file is a simple text file that describes your submission metadata (assembly name, organism, sample accession, etc.) and the path to the gzipped flatfile. The ENA documentation provides template examples.

Step 4: Validate submission using Webin-CLI

The `webin-CLI` tool can be used to validate your files before submission using the following command where `-validate` run validation without submission, `context genome` specifies that this is a genome submission, `-manifest` specifies the path to the manifest file, `-outputdir` specifies the directory where validation reports will be saved, and `userName` and `password` are the ENA login credentials.

```
java -jar webin-cli-8.2.0.jar -validate -context genome -manifest /path/to/manifest_01.txt
 -outputdir /path/to/webin/01 -userName Webin-0000 -password ena-webin-password
```

If validation passes without errors, you can remove -validate to proceed with submission. Errors will be detailed in the .report files in the output directory.

12.2.2 Submission to NCBI

Documentation: https://www.ncbi.nlm.nih.gov/genbank/genomesubmit/

For submission to NCBI, two main options are available depending on your dataset.

Option 1: Using Funannotate (Recommended if using functional annotations)

If you have already used Funannotate annotate [30], the command produces submission-ready .tbl and .sqn files compatible with NCBI requirements (see section 11.4). You can submit these files directly via the NCBI Genome Submission Portal.

Option 2: Using GAG + table2asn

If you prefer not to use Funannotate or only have structural annotation (GFF3), you can use the GAG toolkit [46] to generate submission-ready files (https://genomeannotation.github.io/GAG/).

Step 1: Create .TBL file using GAG

Basic command to use GAG, which produces a .tbl file in the output folder. `--fasta` specifies path to genome assembly file, `--gff` specifies path to cleaned annotation file, `--out` specifies output directory for GAG results.

```
python3 gag.py --fasta Victoria_cruziana.genome.fasta \
 --gff Victoria_cruziana_standard.gff3 \
 --out gag_output
```

Step 2 Convert .TBL to .SQN using table2asn

Table2asn (https://www.ncbi.nlm.nih.gov/genbank/table2asn/) converts the .tbl and genome FASTA into an NCBI submission-ready .sqn format.

Download table2asn:

```
# to get a local copy of table2asn
wget https://ftp.ncbi.nlm.nih.gov/asn1-converters/by_program/table2asn/linux64.table2asn.gz
gunzip linux64.table2asn.gz
mv linux64.table2asn table2asn
chmod +x table2asn
```

table2asn can be run using the following command where `-i` is the input directory with FASTA and TBL files, `-o` is the output directory, `-t` is the submission template (SBT file from NCBI), `-Z` generates error reports, and `-euk` specifies eukaryotic genome.

```
./table2asn -i /path/to/gag_output/ \
 -o /path/to/table2asn_output/ \
 -t SBT_template.txt \
 -M n \
 -Z -euk
```

In the output folder, `.stats` is the summary statistics file, `.val` is the detailed file of errors, and `.dr` is the discrepancy report which shows critical errors. Based on the errors encountered, GAG includes a number of options to remove questionable features like removing terminal N's, fixing start and stop codons, removing short introns etc. Please refer to the official documentation for the full details. Fix errors by adjusting GFF3/FASTA files, rerun GAG and table2asn until no major errors remain.

Step 3: Final submission

Once you obtain a valid .sqn file, submission is done through the NCBI Genome Submission Portal using your NCBI account (https://submit.ncbi.nlm.nih.gov/subs/genome/).

References

1. Merkel D. Docker: Lightweight linux containers for consistent development and deployment. Linux journal. 2014;2014(239):2.
2. Nowak MS, Harder B, Meckoni SN, Friedhoff R, Wolff K, Pucker B. Genome sequence and RNA-seq analysis reveal genetic basis of flower coloration in the giant water lily victoria cruziana. bioRxiv [Internet]. 2025; Available from: https://www.biorxiv.org/content/early/2025/07/22/2024.06.15.599162

3. Katz K, Shutov O, Lapoint R, Kimelman M, Brister JR, O'Sullivan C. The sequence read archive: A decade more of explosive growth. Nucleic Acids Research. 2022 Jan;50(D1):D387–90.

4. Leinonen R, Akhtar R, Birney E, Bower L, Cerdeno-Tárraga A, Cheng Y, Cleland I, Faruque N, Goodgame N, Gibson R, Hoad G, Jang M, Pakseresht N, Plaister S, Radhakrishnan R, Reddy K, Sobhany S, Ten Hoopen P, Vaughan R, Zalunin V, Cochrane G. The european nucleotide archive. Nucleic Acids Research. 2011 Jan;39(Database issue):D28–31.

5. Leinonen R, Sugawara H, Shumway M. The sequence read archive. Nucleic Acids Res [Internet]. 2011 Jan [cited 2023 Jul 21];39:D19–21. Available from: https://www.ncbi.nlm.nih.gov/pmc/articles/PMC3013647/

6. Pellicer J, Leitch IJ. The plant DNA c-values database (release 7.1): An updated online repository of plant genome size data for comparative studies. New Phytologist. 2020;226(2):301–5.

7. Oxford Nanopore Technologies; 2025. Available from: https://github.com/nanoporetech/dorado

8. Danecek P, Bonfield JK, Liddle J, Marshall J, Ohan V, Pollard MO, Whitwham A, Keane T, McCarthy SA, Davies RM, Li H. Twelve years of SAMtools and BCFtools. GigaScience [Internet]. 2021 Jan;10(2). Available from: https://dx.doi.org/10.1093/gigascience/giab008

9. Shen W, Sipos B, Zhao L. SeqKit2: A swiss army knife for sequence and alignment processing. iMeta. 2024;3(3):e191.

10. Stanojević D, Lin D, Nurk S, Sessions PF de, Šikić M. Telomere-to-telomere phased genome assembly using HERRO-corrected simplex nanopore reads. 2024 Oct;2024.05.18.594796. Available from: https://www.biorxiv.org/content/10.1101/2024.05.18.594796v2

11. Shafin K, Pesout T, Lorig-Roach R, Haukness M, Olsen HE, Bosworth C, Armstrong J, Tigyi K, Maurer N, Koren S, Sedlazeck FJ, Marschall T, Mayes S, Costa V, Zook JM, Liu KJ, Kilburn D, Sorensen M, Munson KM, Vollger MR, Monlong J, Garrison E, Eichler EE, Salama S, Haussler D, Green RE, Akeson M, Phillippy A, Miga KH, Carnevali P, Jain M, Paten B. Nanopore sequencing and the shasta toolkit enable efficient de novo assembly of eleven human genomes. Nat Biotechnol [Internet]. 2020 Sep [cited 2023 Jan 11];38(9):1044–53. Available from: https://www.nature.com/articles/s41587-020-0503-6

12. Hu J, Wang Z, Sun Z, Hu B, Ayoola AO, Liang F, Li J, Sandoval JR, Cooper DN, Ye K, Ruan J, Xiao CL, Wang D, Wu DD, Wang S. NextDenovo: An efficient error correction and accurate assembly tool for noisy long reads. Genome Biol [Internet]. 2024 Dec [cited 2025 Jul 24];25(1):1–9. Available from: https://genomebiology.biomedcentral.com/articles/10.1186/s13059-024-03252-4

13. Antipov D, Rautiainen M, Nurk S, Walenz BP, Solar SJ, Phillippy AM, Koren S. Verkko2 integrates proximity-ligation data with long-read de bruijn graphs for efficient telomere-to-telomere genome assembly, phasing, and scaffolding. Genome Res [Internet]. 2025 Jul 1 [cited 2025 Jul 24];35(7):1583–94. Available from: http://genome.cshlp.org/content/35/7/1583

14. Cheng H, Concepcion GT, Feng X, Zhang H, Li H. Haplotype-resolved de novo assembly using phased assembly graphs with hifiasm. Nat Methods [Internet]. 2021 Feb [cited 2025 Jul 24];18(2):170–5. Available from: https://www.nature.com/articles/s41592-020-01056-5

15. Manni M, Berkeley MR, Seppey M, Simão FA, Zdobnov EM. BUSCO update: Novel and streamlined workflows along with broader and deeper phylogenetic coverage for scoring of eukaryotic, prokaryotic, and viral genomes. Molecular Biology and Evolution [Internet]. 2021 Oct 1 [cited 2025 Jul 24];38(10):4647–54. Available from: https://doi.org/10.1093/molbev/msab199

16. Tegenfeldt F, Kuznetsov D, Manni M, Berkeley M, Zdobnov EM, Kriventseva EV. OrthoDB and BUSCO update: Annotation of orthologs with wider sampling of genomes. Nucleic Acids Research [Internet]. 2025 Jan 6 [cited 2025 Jul 25];53:D516–22. Available from: https://academic.oup.com/nar/article/53/D1/D516/7899526

17. Ou S, Jiang N. LTR_retriever: A highly accurate and sensitive program for identification of long terminal repeat retrotransposons. Plant Physiol [Internet]. 2018 Feb [cited 2025 Jul 25];176(2):1410–22. Available from: https://academic.oup.com/plphys/article/176/2/1410-1422/6117145

18. Ou S, Chen J, Jiang N. Assessing genome assembly quality using the LTR assembly index (LAI). Nucleic Acids Research [Internet]. 2018 Aug 10 [cited 2025 Jul 25]; Available from: https://academic.oup.com/nar/advance-article/doi/10.1093/nar/gky730/5068908

19. Natarajan S, Gehrke J, Pucker B. Mapping-based genome size estimation. BMC Genomics [Internet]. 2025 May 14 [cited 2025 Jul 25];26(1). Available from: https://bmcgenomics.biomedcentral.com/articles/10.1186/s12864-025-11640-8

20. Rhie A, Walenz BP, Koren S, Phillippy AM. Merqury: Reference-free quality, completeness, and phasing assessment for genome assemblies. Genome Biol [Internet]. 2020 Dec [cited 2025 Jul 25];21(1). Available from: https://genomebiology.biomedcentral.com/articles/10.1186/s13059-020-02134-9

21. Ewing B, Green P. Base-calling of automated sequencer traces using phred. II. Error probabilities. Genome Research. 1998 Mar;8(3):186–94.

22. Mikheenko A, Saveliev V, Hirsch P, Gurevich A. WebQUAST: Online evaluation of genome assemblies. Nucleic Acids Research [Internet]. 2023 Jul 5 [cited 2025 Jul 25];51:W601–6. Available from: https://academic.oup.com/nar/article/51/W1/W601/7167303

23. Zhang Y, Park C, Bennett C, Thornton M, Kim D. Rapid and accurate alignment of nucleotide conversion sequencing reads with HISAT-3N. Genome Res [Internet]. 2021 Jul [cited 2025 Jul 25];31(7):1290–5. Available from: http://genome.cshlp.org/lookup/doi/10.1101/gr.275193.120

24. Dobin A, Davis CA, Schlesinger F, Drenkow J, Zaleski C, Jha S, Batut P, Chaisson M, Gingeras TR. STAR: Ultrafast universal RNA-seq aligner. Bioinformatics [Internet]. 2013 Jan 1 [cited 2025 Jul 25];29(1):15–21. Available from: https://academic.oup.com/bioinformatics/article/29/1/15/272537

25. Gabriel L, Brůna T, Hoff KJ, Ebel M, Lomsadze A, Borodovsky M, Stanke M. BRAKER3: Fully automated genome annotation using RNA-seq and protein evidence with GeneMark-ETP, AUGUSTUS, and TSEBRA. Genome Res [Internet]. 2024 May [cited 2025 Jul 25];34(5):769–77. Available from: http://genome.cshlp.org/lookup/doi/10.1101/gr.278090.123

26. Brůna T, Lomsadze A, Borodovsky M. GeneMark-ETP significantly improves the accuracy of automatic annotation of large eukaryotic genomes. Genome Research. 2024 May;34(5):757–68.

27. Stanke M, Diekhans M, Baertsch R, Haussler D. Using native and syntenically mapped cDNA alignments to improve de novo gene finding. Bioinformatics. 2008 Mar;24(5):637–44.

28. The UniProt Consortium, Bateman A, Martin MJ, Orchard S, Magrane M, Adesina A, Ahmad S, Bowler-Barnett EH, Bye-A-Jee H, Carpentier D, Denny P, Fan J, Garmiri P, Gonzales LJDC, Hussein A, Ignatchenko A, Insana G, Ishtiaq R, Joshi V, Jyothi D, Kandasaamy S, Lock A, Luciani A, Luo J, Lussi Y, Marin JSM, Raposo P, Rice DL, Santos R, Speretta E, Stephenson J, Totoo P, Tyagi N, Urakova N, Vasudev P, Warner K, Wijerathne S, Yu CWH, Zaru R, Bridge AJ, Aimo L, Argoud-Puy G, Auchincloss AH, Axelsen KB, Bansal P, Baratin D, Batista Neto TM, Blatter MC, Bolleman JT, Boutet E, Breuza L, Gil BC, Casals-Casas C, Echioukh KC, Coudert E, Cuche B, De Castro E, Estreicher A, Famiglietti ML, Feuermann M, Gasteiger E, Gaudet P, Gehant S, Gerritsen V, Gos A, Gruaz N, Hulo C, Hyka-Nouspikel N, Jungo F, Kerhornou A, Mercier PL, Lieberherr D, Masson P, Morgat A, Paesano S, Pedruzzi I, Pilbout S, Pourcel L, Poux S, Pozzato M, Pruess M, Redaschi N, Rivoire C, Sigrist CJA, Sonesson K, Sundaram S, Sveshnikova A, Wu CH, Arighi CN, Chen C, Chen Y, Huang H, Laiho K, Lehvaslaiho M, McGarvey P, Natale DA, Ross K, Vinayaka CR, Wang Y, Zhang J. UniProt: The universal protein knowledgebase in 2025. Nucleic Acids Research [Internet]. 2025 Jan 6 [cited 2025 Jul 25];53:D609–17. Available from: https://academic.oup.com/nar/article/53/D1/D609/7902999

29. Keilwagen J, Hartung F, Grau J. GeMoMa: Homology-based gene prediction utilizing intron position conservation and RNA-seq data. In: Kollmar M, editor. Gene prediction: Methods and protocols [Internet]. New York, NY: Springer; 2019 [cited 2024 Jul 8]. p. 161–77. Available from: https://doi.org/10.1007/978-1-4939-9173-0_9

30. Palmer JM, Stajich J. Funannotate v1.8.1: Eukaryotic genome annotation. 2020 Sep 28 [cited 2025 Jul 25]; Available from: https://zenodo.org/record/4054262

31. Holst F, Bolger AM, Kindel F, Günther C, Maß J, Triesch S, Kiel N, Saadat N, Ebenhöh O, Usadel B, Schwacke R, Weber APM, Bolger ME, Denton AK. Helixer: Ab initio prediction of primary eukaryotic gene models combining deep learning and a hidden markov model. Nat Methods [Internet]. 2025 Nov 24 [cited 2025 Dec 9];1–8. Available from: https://www.nature.com/articles/s41592-025-02939-1

32. Gabriel L, Becker F, Hoff KJ, Stanke M. Tiberius: End-to-end deep learning with an HMM for gene prediction. Bioinformatics [Internet]. 2024 Dec 1 [cited 2025 Dec 9];40(12):btae685. Available from: https://doi.org/10.1093/bioinformatics/btae685

33. Ou S, Su W, Liao Y, Chougule K, Agda JRA, Hellinga AJ, Lugo CSB, Elliott TA, Ware D, Peterson T, Jiang N, Hirsch CN, Hufford MB. Benchmarking transposable element annotation methods for creation of a streamlined, comprehensive pipeline. Genome Biol [Internet]. 2019 Dec 16 [cited 2025 Jul 25];20(1). Available from: https://genomebiology.biomedcentral.com/articles/10.1186/s13059-019-1905-y

34. Nawrocki EP, Eddy SR. Infernal 1.1: 100-fold faster RNA homology searches. Bioinformatics [Internet]. 2013 Nov 15 [cited 2025 Jul 25];29(22):2933–5. Available from: https://academic.oup.com/bioinformatics/article/29/22/2933/316439

35. Ontiveros-Palacios N, Cooke E, Nawrocki EP, Triebel S, Marz M, Rivas E, Griffiths-Jones S, Petrov AI, Bateman A, Sweeney B. Rfam 15: RNA families database in 2025. Nucleic Acids Research [Internet]. 2025 Jan 6 [cited 2025 Jul 25];53:D258–67. Available from: https://academic.oup.com/nar/article/53/D1/D258/7889252

36. Chan PP, Lin BY, Mak AJ, Lowe TM. tRNAscan-SE 2.0: Improved detection and functional classification of transfer RNA genes. Nucleic Acids Research. 2021;49(16):9077–96.

37. Lagesen K, Hallin P, Rødland EA, Stærfeldt HH, Rognes T, Ussery DW. RNAmmer: Consistent and rapid annotation of ribosomal RNA genes. Nucleic Acids Research. 2007 May;35(9):3100–8.

38. Nawrocki EP. STRUCTURAL RNA HOMOLOGY SEARCH AND ALIGNMENT USING COVARIANCE MODELS.

39. Cheng CY, Krishnakumar V, Chan AP, Thibaud-Nissen F, Schobel S, Town CD. Araport11: A complete reannotation of the arabidopsis thaliana reference genome. The Plant Journal. 2017;89(4):789–804.

40. Jones P, Binns D, Chang HY, Fraser M, Li W, McAnulla C, McWilliam H, Maslen J, Mitchell A, Nuka G, Pesseat S, Quinn AF, Sangrador-Vegas A, Scheremetjew M, Yong SY, Lopez R, Hunter S. InterProScan 5: Genome-scale protein function classification. Bioinformatics [Internet]. 2014 May 1 [cited 2025 Jul 25];30(9):1236–40. Available from: https://academic.oup.com/bioinformatics/article/30/9/1236/237988

41. Rempel A, Choudhary N, Pucker B. KIPEs3: Automatic annotation of biosynthesis pathways. Ezura H, editor. PLoS ONE [Internet]. 2023 Nov 16 [cited 2025 Jul 25];18(11):e0294342. Available from: https://dx.plos.org/10.1371/journal.pone.0294342

42. Pucker B. Automatic identification and annotation of MYB gene family members in plants. BMC Genomics [Internet]. 2022 Dec [cited 2025 Jul 25];23(1). Available from: https://bmcgenomics.biomedcentral.com/articles/10.1186/s12864-022-08452-5

43. Thoben C, Pucker B. Automatic annotation of the bHLH gene family in plants. BMC Genomics [Internet]. 2023 Dec 15 [cited 2025 Jul 25];24(1). Available from: https://bmcgenomics.biomedcentral.com/articles/10.1186/s12864-023-09877-2

44. Dainat J. Another gtf/gff analysis toolkit (AGAT): Resolve interoperability issues and accomplish more with your annotations. Plant and animal genome XXIX conference. 2022; Available from: https://github.com/NBISweden/AGAT

45. Norling M, Jareborg N, Dainat J. EMBLmyGFF3: A converter facilitating genome annotation submission to european nucleotide archive. BMC Res Notes [Internet]. 2018 Dec [cited 2025 Jul 25];11(1). Available from: https://bmcresnotes.biomedcentral.com/articles/10.1186/s13104-018-3686-x

46. Hall B, DeRego T, Geib S. GAG: The genome annotation generator (version 1.0). 2014; Available from: http://genomeannotation.github.io/GAG/
